# Supplementary material for: Learning and attention increase visual response selectivity through distinct mechanisms
Source: Neuron. 2022 Feb 16;110(4):686–697.e6. doi: 10.1016/j.neuron.2021.11.016 (PMC8860382; doi:10.1016/j.neuron.2021.11.016)
Supplement: Document S2. Article plus supplemental information [file mmc2.pdf]

Article

Neuron

Learning and attention increase visual response selectivity through distinct mechanisms

Highlights

- Learning and attention both enhance sensory processing at different timescales
- The effects of learning and attention are uncorrelated at the single-cell level
- Learning is driven by response suppression, attention by enhancement and suppression
- Model with cell class-specific top-down inputs can account for effects of attention

Authors

Jasper Poort, Katharina A. Wilmes, Antonin Blot, ..., Thomas D. Mrsic-Flogel, Sonja B. Hofer, Adil G. Khan

Correspondence

jp816@cam.ac.uk (J.P.), khan.adil@kcl.ac.uk (A.G.K.)

In brief

Learning and attention can enhance sensory processing in cortical circuits at longer and shorter timescales. By tracking the same excitatory and inhibitory neurons in the visual cortex across learning and during attention switching, Poort et al. demonstrate that distinct mechanisms underlie the enhancement of sensory processing due to learning and attention.

Poort et al., 2022, Neuron *110*, 686–697
February 16, 2022 © 2021 The Authors. Published by Elsevier Inc.
https://doi.org/10.1016/j.neuron.2021.11.016

CellPress

CellPress
OPEN ACCESS

Neuron

Article

Learning and attention increase visual response selectivity through distinct mechanisms

Jasper Poort,[1,2,8,9,*] Katharina A. Wilmes,[3] Antonin Blot,[4,5] Angus Chadwick,[6] Maneesh Sahani,[6] Claudia Clopath,[3] Thomas D. Mrsic-Flogel,[4,5] Sonja B. Hofer,[4,5] and Adil G. Khan[4,7,8,*]
[1]Department of Physiology, Development and Neuroscience, University of Cambridge, Cambridge, UK
[2]Department of Psychology, University of Cambridge, Cambridge, UK
[3]Department of Bioengineering, Imperial College, London, UK
[4]Biozentrum, University of Basel, Basel, Switzerland
[5]Sainsbury Wellcome Centre for Neural Circuits and Behavior, University College London, London, UK
[6]Gatsby Computational Neuroscience Unit, University College London, London, UK
[7]Centre for Developmental Neurobiology, King's College London, London, UK
[8]These authors contributed equally
[9]Lead author
*Correspondence: jp816@cam.ac.uk (J.P.), khan.adil@kcl.ac.uk (A.G.K.)
https://doi.org/10.1016/j.neuron.2021.11.016

SUMMARY

Selectivity of cortical neurons for sensory stimuli can increase across days as animals learn their behavioral relevance and across seconds when animals switch attention. While both phenomena occur in the same circuit, it is unknown whether they rely on similar mechanisms. We imaged primary visual cortex as mice learned a visual discrimination task and subsequently performed an attention switching task. Selectivity changes due to learning and attention were uncorrelated in individual neurons. Selectivity increases after learning mainly arose from selective suppression of responses to one of the stimuli but from selective enhancement and suppression during attention. Learning and attention differentially affected interactions between excitatory and PV, SOM, and VIP inhibitory cells. Circuit modeling revealed that cell class-specific top-down inputs best explained attentional modulation, while reorganization of local functional connectivity accounted for learning-related changes. Thus, distinct mechanisms underlie increased discriminability of relevant sensory stimuli across longer and shorter timescales.

INTRODUCTION

Learning and attention both selectively enhance the processing of behaviorally relevant stimuli (Gdalyahu et al., 2012; Goltstein et al., 2013; Li et al., 2008; McAdams and Maunsell, 1999; Ni et al., 2018; Reynolds and Chelazzi, 2004; Rutkowski and Weinberger, 2005; Schoups et al., 2001; Speed et al., 2020; Wiest et al., 2010; Yan et al., 2014; Yang and Maunsell, 2004). When animals learn what sensory features are task relevant or when they focus their attention on task-relevant features, early sensory cortical representations often undergo substantial changes. However, it is not known whether cortical changes during learning and attention rely on similar neural mechanisms.

The neural correlates of learning and attention share several characteristics. Visual learning results in increased stimulus selectivity through changes in stimulus-evoked neural firing rates (Gilbert and Li, 2012; Karmarkar and Dan, 2006; Li et al., 2008; Poort et al., 2015; Schoups et al., 2001; Yan et al., 2014; Yang and Maunsell, 2004), and is accompanied by changes in the interactions and correlations between neurons (Gu et al., 2011; Khan et al., 2018; Ni et al., 2018). Similarly, visual attention can also result in increased selectivity of attended stimuli, again through changes in stimulus-evoked firing rates (Reynolds and Chelazzi, 2004; Speed et al., 2020; Spitzer et al., 1988; Wimmer et al., 2015) and neuronal interactions (Cohen and Maunsell, 2009; Mitchell et al., 2009; Ni et al., 2018). Importantly, activity modulations during learning and attention are not uniformly distributed throughout the neural population but are restricted to subsets of neurons (see, for example, Chen et al., 2008; McAdams and Maunsell, 1999; Poort et al., 2015; Schoups et al., 2001; Yan et al., 2014). Thus, both learning and attention lead to sharper and more distinct information being sent to downstream regions though subnetworks of learning- or attention-modulated cells.

Inhibition plays a crucial role in cortical plasticity (Froemke, 2015; van Versendaal and Levelt, 2016), and specific classes of inhibitory interneurons have been implicated in the plasticity of cortical circuits during both learning and attention (Chen et al., 2015; Kato et al., 2015; Kuchibhotla et al., 2017; Makino and Komiyama, 2015; Sachidhanandam et al., 2016; Yazaki-Sugiyama et al., 2009). The activity of interneurons can change during both learning (Kato et al., 2015; Khan et al., 2018; Letzkus

 © 2021 The Authors. Published by Elsevier Inc.
This is an open access article under the CC BY license (http://creativecommons.org/licenses/by/4.0/).

CellPress
OPEN ACCESS

et al., 2011; Makino and Komiyama, 2015) and attention (Mitchell et al., 2007; Snyder et al., 2016; Speed et al., 2020), which can result in more stimulus-specific inhibition in the network.

Both learning and attention rely, to varying degrees, on the integration of top-down inputs with bottom-up signals. During attention, higher-order brain regions are thought to provide feedback signals to bias bottom-up information processing (Desimone and Duncan, 1995; Gilbert and Li, 2013), most prominently through direct feedback projections (Leinweber et al., 2017; Zhang et al., 2014) or through thalamic nuclei (Chalupa et al., 1976; Wimmer et al., 2015). These feedback projections can target excitatory or specific inhibitory interneurons (Leinweber et al., 2017; Zhang et al., 2014, 2016). In contrast, learning is thought to be primarily implemented by long-term plasticity of synapses, and reorganization of connectivity patterns (Froemke, 2015; Khan et al., 2018; Whitlock et al., 2006; Xiong et al., 2015), although top-down projections may also play a crucial role in guiding this process (Roelfsema and Holtmaat, 2018; Williams and Holtmaat, 2019).

Thus, both learning and attention modulate the firing properties of subsets of excitatory and inhibitory cortical neurons, leading to changes in firing rates and interactions between cells. It has therefore been suggested that learning and attention rely on similar neural mechanisms (Ni et al., 2018) or that attention-like processes may co-opt some of the underlying circuitry of learning (Kuchibhotla et al., 2017). However, this has never directly been tested, and it is not known whether learning and attention engage the same neurons and circuits. A number of questions thus arise. First, within a population, is a common subset of neurons modulated by both learning and attention? Second, do learning-modulated and attention-modulated neurons undergo similar changes in their firing rates to increase stimulus selectivity? Third, do learning and attention result in similar changes in interactions between different excitatory and inhibitory cell classes?

To address these questions, we compared the changes in activity and interactions of the same population of neurons in V1 during learning and attention. We tracked the same identified pyramidal (PYR) neurons and parvalbumin- (PV), somatostatin- (SOM), and vasoactive intestinal peptide (VIP)-positive interneurons as mice learned to discriminate two visual stimuli and subsequently performed an attention-switching task involving the same visual stimuli. We observed a similar profile of average changes in stimulus selectivity across the four cell classes during learning and attention. However, we discovered that these changes were uncorrelated at the single-cell level, consistent with distinct mechanisms of selectivity changes during learning and attention. In support of this idea, we found that neural stimulus responses were dominated by selective suppression during learning, but displayed a combination of suppression and enhancement during attention. In addition, learning and attention differentially modulated interactions between excitatory and inhibitory cell classes. While learning-related changes were well captured by a model invoking changes in functional interaction strengths, attention-related changes were captured by a circuit model with top-down inputs targeted to PYR and SOM cells. These results reveal that more selective cortical representations for behaviorally relevant stimuli arise through distinct mechanisms over longer and shorter timescales.

RESULTS

Increased response selectivity related to learning and attention switching

To understand how the same neural populations change their responses to visual stimuli with learning and attention, we trained mice to learn a go-no go visual discrimination task and subsequently trained them to perform an attention-switching task involving the same pair of visual stimuli (Figures 1A and 1B). Head-fixed mice ran through a virtual approach corridor (Figure 1A) where the walls displayed a short stretch of circle patterns followed by gray walls for a random distance chosen from an exponential distribution (Figure 1C, top). Mice were then presented with one of two grating patterns, vertical or angled (40° relative to vertical), and were rewarded for licking a reward spout in response to the vertical grating. No punishment was given for licking the spout in response to angled gratings. All mice learned to discriminate the grating stimuli, reaching a threshold criterion of $d' > 2.0$ (~85% accuracy) within 7–9 days (Figure S1 example lick rasters from sessions pre- and post-learning; Figure 1D, average behavioral d′ pre-learning −0.18 ± 0.56 SD, post-learning 3.32 ± 0.82, sign test, $p = 0.008$, N = 8 mice).

We subsequently trained the mice to switch between blocks of the same visual discrimination task and an olfactory discrimination task, in which they learned to lick the reward spout to obtain a reward in response to one of two odors. During the olfactory discrimination blocks, the same grating stimuli used in the visual discrimination blocks were presented on 70% of trials but were irrelevant to the task (Figure 1C, bottom). Mice learned this attention-switching task in 1–2 days. Mice switched between the two blocks within the same session, successfully attending to and discriminating the grating stimuli in the visual block but ignoring the same grating stimuli while successfully discriminating odors during the olfactory blocks (Figure S1 example lick rasters from a session of attention-switching behavior; Figure 1D, behavioral d′ attend visual 3.02 ± 0.41 versus ignore visual 0.63 ± 0.25, sign test $p = 0.015$, d′ discriminating olfactory stimuli 4.10 ± 0.27).

Selectivity changes at the population level are similar across learning and attention

We expressed the calcium indicator GCaMP6f in V1 using viral vectors and measured responses of layer 2/3 neurons using two-photon calcium imaging during the task. We re-identified the same neurons in co-registered, immunohistochemically stained brain sections from these animals and determined the identity of putative excitatory PYR neurons and cells belonging to the three major classes of GABAergic inhibitory interneurons (Figure 2A). This approach allowed us to measure the simultaneous activity of PV-, SOM-, and VIP- positive interneurons along with the local excitatory neuron population (see Method details). We imaged the same 1,848 PYR, 193 PV, 78 SOM, and 237 VIP neurons before and after learning and a partially overlapping population of 6,013 PYR, 596 PV, 263 SOM, and 366 VIP neurons during the attention-switching task (1,469, 166, 74, and 198 cells overlapping respectively, N = 9 mice; all four cell classes were identified in all mice;

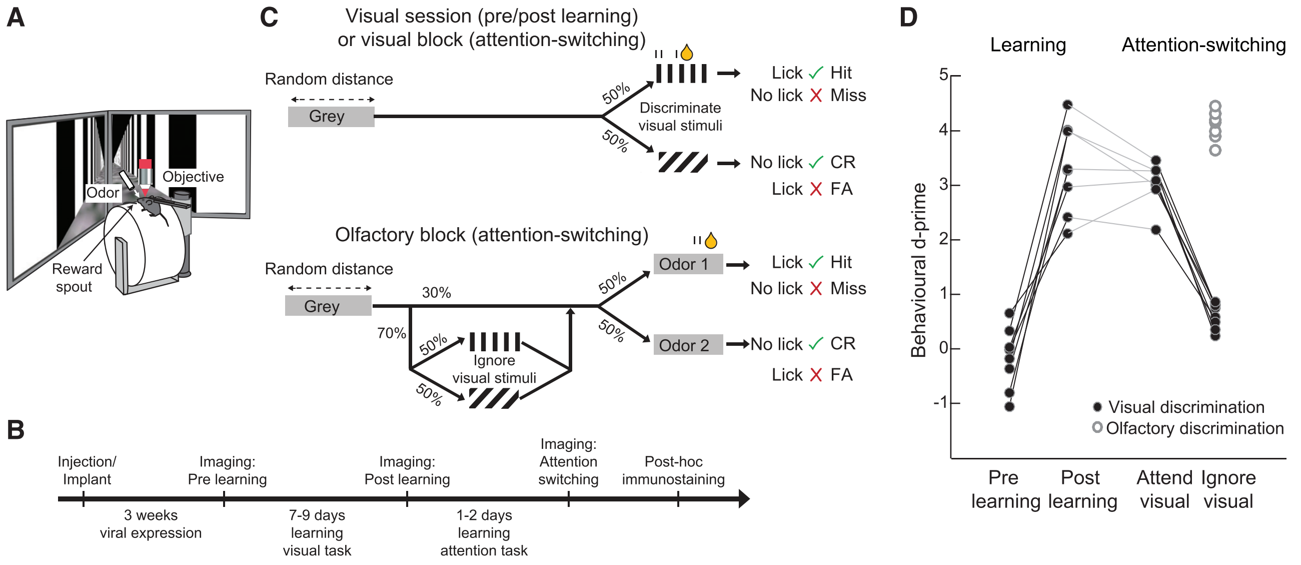

Figure 1. Visual discrimination learning and attention switching in mice
(A) Top, schematic showing virtual reality and imaging setup.
(B) Experimental timeline.
(C) Schematic of behavioral tasks. Top, visual discrimination: mice were rewarded for licking the reward spout when vertical gratings were presented and not when angled gratings were presented. Olfactory discrimination: mice were rewarded for licking when odor 1 was presented and not when odor 2 or vertical or angled gratings were presented.
(D) Behavioral discrimination performance (behavioral d′) across learning and during attention switching (N = 9 mice, 7 of which were tracked across both learning and attention). Connected closed points indicate visual discrimination in individual mice. Open circles indicate olfactory discrimination.
See also Figure S1.

see Figure S2 for distribution of cells across mice and cell type).

Neurons from each cell class showed varying degrees of responsiveness to the visual grating stimuli (Figures S3A and S3B). During learning, we observed changes in visual grating responses in subsets of neurons from all cell classes (Figures 2B, S3A, and S3B). This led to changes in stimulus selectivity (difference in the mean responses to the two grating stimuli normalized by response variability; see Method details) in individual cells to varying degrees (Figure 2C). On average, PYR and PV cells significantly increased their stimulus selectivity during learning, as reported previously (Khan et al., 2018; Poort et al., 2015) (Figure 2D; PYR, average absolute selectivity pre-learning, 0.27 ± 0.28 [mean ± SD], post-learning 0.37 ± 0.39, sign test, $p = 2 \times 10^{-10}$, N = 1,469; PV, pre-learning, 0.22 ± 0.18, post-learning 0.38 ± 0.34, $p = 2 \times 10^{-5}$, N = 166). In contrast, the average selectivity of SOM and VIP interneurons did not change significantly (SOM, pre-learning 0.24 ± 0.16, post-learning 0.32 ± 0.34, p = 0.91, N = 74; VIP, pre-learning 0.17 ± 0.13, post-learning 0.20 ± 0.18, p = 0.62, N = 198).

We found a similar profile of selectivity changes across cell classes between the “ignore” and “attend” conditions of the attention-switching task. Specifically, visual stimulus selectivity increased on average in PYR and PV cells but not in SOM and VIP cells when mice switched from ignoring to attending the same visual grating stimuli (Figures 2E–2G; PYR, ignore 0.30 ± 0.30, attend 0.39 ± 0.37, $p = 9 \times 10^{-13}$, N = 1,469; PV, ignore 0.26 ± 0.19, attend 0.35 ± 0.29, p = 0.0008, N = 166; SOM, ignore 0.35 ± 0.38, attend 0.30 ± 0.34, p = 0.30, N = 74; VIP, ignore 0.25 ± 0.18, attend 0.26 ± 0.18, p = 0.62, N = 198; data from the same cells matched across learning and attention). Changes in running and licking could not account for the increased selectivity of responses during learning or attention (Figures S4A and S4B; see also Figure S2A for data from individual mice). Thus, learning and attention both led to similar changes in stimulus selectivity of V1 neurons on average, across excitatory and multiple inhibitory cell classes.

Selectivity changes at the single-cell level are uncorrelated

The similar profile of changes in average selectivity during learning and attention switching suggested that the neural basis of these two changes may overlap. Both learning and attention serve a similar purpose: to enhance the ability of an animal to detect and respond to relevant stimuli, and prior work has suggested that the two may be implemented by common neural mechanisms (Ni et al., 2018). We therefore asked whether the increase in selectivity during learning and attention was related at the single-neuron level.

Across the population of PYR neurons that were identified across both learning and attention, we found that there was no significant correlation between the learning-related and attention-related changes in stimulus selectivity (Figure 3A; R = 0.03, p = 0.25; see also Figure S3C). This indicated that the change in stimulus selectivity of a cell during learning had no bearing on its change during attention. This absence of correlation was not due to extensive changes in the original visual response selectivity of these cells from the post-learning session

CellPress
OPEN ACCESS

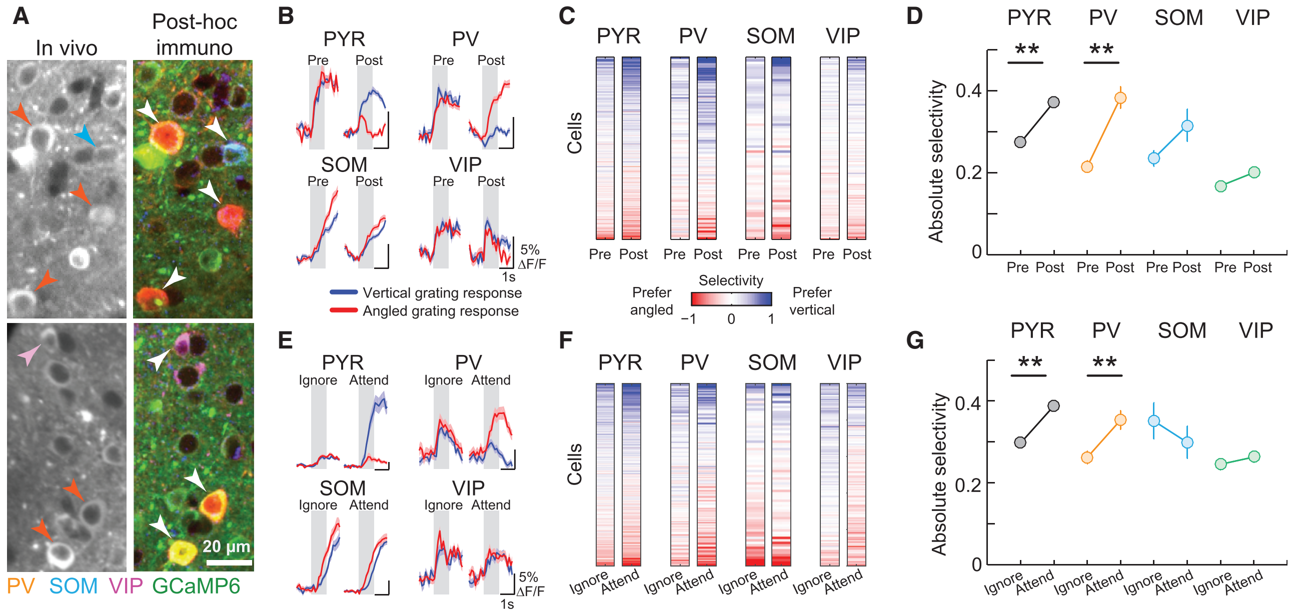

Figure 2. Similar changes in stimulus response selectivity across 4 cell classes during learning and attention switching

(A) Two example regions of *in vivo* image planes with GCaMP6f-expressing neurons and the same regions after post hoc immunostaining for PV, SOM, and VIP (orange, blue, and magenta, respectively) following image registration. Identified interneurons are indicated by arrowheads.

(B) Example cells from the 4 cell classes, average responses to vertical (blue line), and angled (red line) grating stimuli before (pre) and after (post) learning. Shaded area represents SEM. Gray shading indicates 0–1 s window from stimulus onset used to calculate stimulus selectivity.

(C) Stimulus selectivity of the same cells (rows) before and after learning (columns). Cells were ordered by their mean pre- and post-learning selectivity.

(D) Average absolute selectivity of the 4 cell classes before and after learning. Error bars represent SEMs. Sign test, $^{**}p < 0.001$. Selectivity distribution in Figure S5A.

(E–G) Same as (B)–(D) for attention-switching task.

Cells in (C), (D), (F), and (G) were tracked both pre- and post-learning and during the attention task. N = 1,469 PYR, 166 PV, 74 SOM, and 198 VIP cells. See also Figures S2, S4, and S5.

to the attention-switching session; there was a strong correlation between the post-learning selectivity and the selectivity during the attend condition of the attention-switching task (Figure 3B; $R = 0.53$, $p = 2.6 \times 10^{-108}$).

Similarly, we observed no correlation between the learning-related and attention-related changes in PV, SOM, or VIP interneurons (Figure 3C; PV, $R = 0.07$, $p = 0.40$; SOM, $R = -0.08$, $p = 0.49$; VIP, $R = -0.11$, $p = 0.13$; see also Figure S2B for data from individual mice). All of the interneuron cell classes also displayed strong correlations between the post-learning selectivity and the selectivity during the attend condition (Figure 3D; PV, $R = 0.52$, $p = 1.1 \times 10^{-12}$; SOM, $R = 0.46$, $p = 3.9 \times 10^{-5}$; VIP, $R = 0.37$, $p = 6.0 \times 10^{-8}$), and all of the cell classes displayed strong correlations between the post-learning selectivity and the selectivity during the ignore condition (R = 0.53, 0.35, 0.51, and 0.25 for PYR, PV, SOM, and VIP cells, respectively; all $p < 10^{-3}$), again ruling out extensive changes in the stimulus tuning of cells between the post-learning and attention-switching sessions.

Thus, while increases in neural selectivity due to learning and attention were similar across excitatory and multiple inhibitory interneuron classes on average, they were uncorrelated at the single-cell level. The lack of correlation between selectivity modulations during learning and attention suggested that these two processes may be driven by distinct neural mechanisms.

Mechanisms of selectivity change

Neurons can increase their stimulus selectivity by selective suppression of responses to non-preferred stimuli (Lee et al., 2012), selective increase in responses to preferred stimuli (McAdams and Maunsell, 1999), or a combination of the two. We tested for the relative prevalence of these changes in the population of PYR cells during learning and attention.

We studied changes in stimulus-evoked firing rates in all recorded PYR cells, regardless of their stimulus selectivity. We subtracted the pre-learning from the post-learning stimulus response profile of each cell for a given stimulus to obtain the difference-peristimulus time histogram (PSTH). During learning, the difference-PSTHs of the PYR population were dominated by cells with negative deflections from baseline—in other words, cells that decreased their stimulus response amplitude to the same stimulus during learning (Figure 4A, left). This was true for both rewarded and non-rewarded stimuli (Figure S6A, left). Interestingly, the difference-PSTH during attention switching (attend minus ignore condition) revealed that changes with attention were more uniformly distributed across increases and decreases in response amplitude (Figure 4A, right). This was again true for both rewarded and non-rewarded stimuli (Figure S6A, right, difference-PSTH averaged 0–1 s significantly different between learning and attention, $p = 0$, sign test; Figure S6D). Thus, learning, unlike attention, was dominated by a suppression of responses.

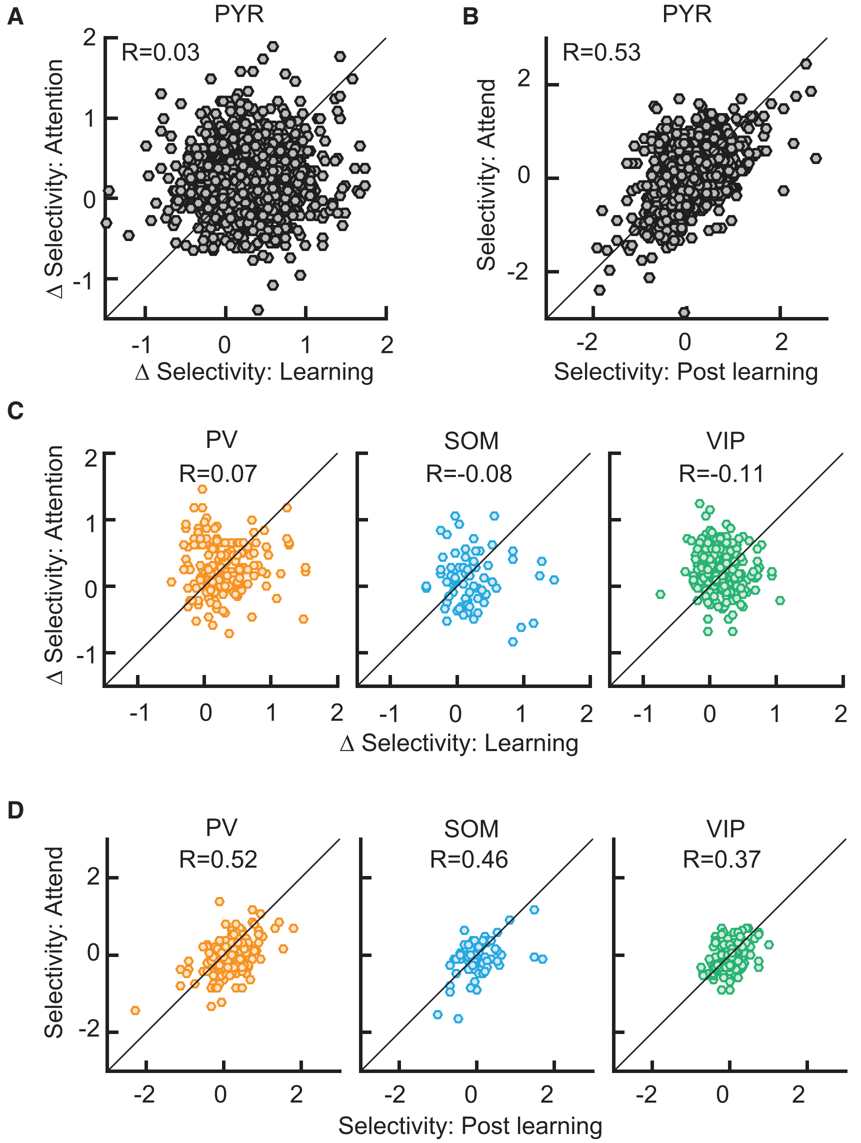

Figure 3. Changes in stimulus selectivity during learning and attention are uncorrelated

(A) Relationship between ΔSelectivity with learning (positive values indicate increased selectivity after learning) and ΔSelectivity with attention (positive values indicate increased selectivity with attention) for PYR cells (N = 1,469 cells).

(B) Relationship between post-learning selectivity and selectivity in the attend condition for PYR cells.

(C and D) Same as (A) and (B) for the 3 interneuron classes (N = 166 PV, 74 SOM, and 198 VIP cells).

See also Figure S3.

Learning and attention may lead to complex temporal changes in firing rate profiles, not captured in the above analysis. We therefore performed principal-component analysis (PCA) to identify the components that captured the majority of variance in the shapes of all difference-PSTHs. Interestingly, for both learning and attention, we found that a single component accounted for >85% of the variance across all cells, and this component had a similar temporal profile for both learning and attention (Figures 4B and 4C). However, the distributions of weights projected onto this PC during learning and attention were substantially different, with a predominance of negative weights during learning (Figure 4D; $p = 0$, sign test). Thus, while we did not find a difference in the temporal profile of firing rate changes, we confirmed the robust presence of stimulus response suppression during learning, but not during attention.

At the single-cell level, we found that the scores of the same neurons on the first PCA components for learning and attention had a low correlation (Figure 4E; $R = 0.12$, $p = 9.7 \times 10^{-6}$; see Figure S6E for a similar effect with average calcium responses), suggesting near-independent firing rate modulation of individual cell responses to the same stimuli by learning and attention.

We next asked what changes in firing rates underlie the increased stimulus selectivity in the population. We restricted this analysis to the subset of cells that changed from non-selective to significantly selective for any stimulus during learning or attention. The average PSTHs of these "recruited" cells showed markedly distinct features. During learning, recruited cells showed preferential suppression of responses to one of the two stimuli (Figure 4F). In contrast, with attention, cells became selective through a combination of enhancement and suppression of responses to the two stimuli (Figure 4G, percentage changes in stimulus response amplitude to vertical and angled stimuli: Figure 4F, left, −12%, −83%; Figure 4F, right −90%, −34%; Figure 4G, left, 69%, 7%, not significant; Figure 4G right −94%, 56%; changes were calculated as the percentage of the maximum in each category; all of the responses averaged 0–1 s and all $p < 10^{-6}$, except where stated).

Thus, learning was associated with suppression of evoked responses, particularly of the non-preferred stimulus, while attention was mainly associated with increased responses of the preferred stimulus.

Changes in interactions between excitatory and inhibitory cell classes

Changes in cortical processing are accompanied by a reconfiguration of network dynamics and interactions. We previously demonstrated that interactions between PV cells and surrounding PYR cells are reorganized during learning (Khan et al., 2018). Specifically, we measured the correlation between PV cell selectivity and the selectivity of the PYR cell population within 100 μm of each PV cell. The slope of the line of best fit and correlation coefficient of this relationship significantly decreased during

CellPress
OPEN ACCESS

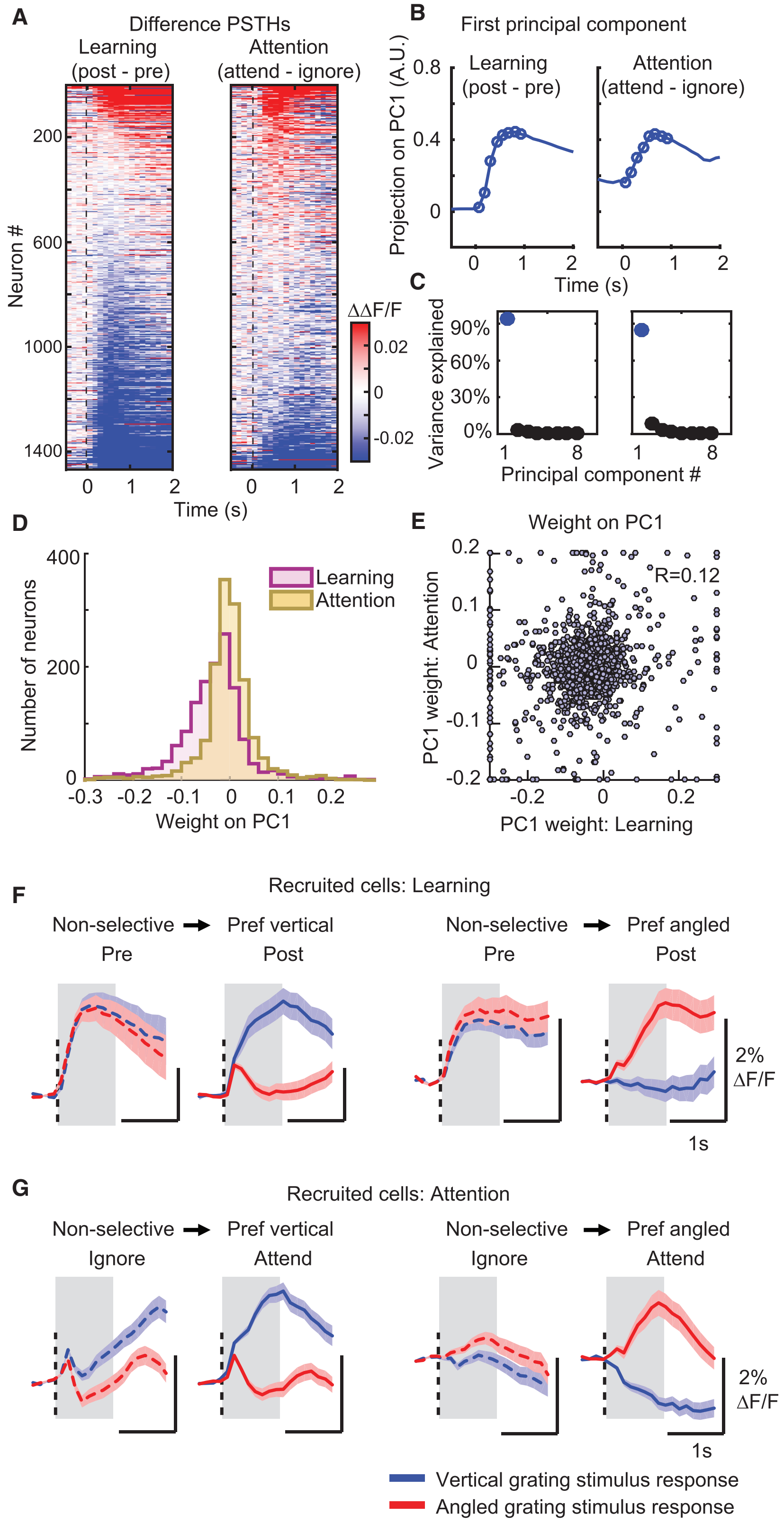

Figure 4. Increased stimulus selectivity through selective response suppression during learning but enhancement and suppression during attention

(A) Difference in calcium responses to the rewarded vertical grating stimulus, post- minus pre-learning (left) or attend minus ignore conditions (right) for all recorded PYR cells (difference-PSTHs). Responses are baseline corrected (subtraction of baseline ΔF/F −0.5 to 0 s before stimulus onset) and aligned to grating onset (dashed line). Cells are sorted by their average amplitude 0–1 s from stimulus onset. N = 1,469 matched PYR cells, in (A)–(E), N = 7 mice.

(B) First principal component (PC) of the difference-PSTHs from the learning (left) and attention data (right). Circles indicate the time points (0–1 s) used to determine the PCs.

(C) Percentage of variance explained by each PC during learning (left) and attention (right).

(D) Distribution of weights from each cell onto the first PC during learning and attention.

(E) Relationship between the weights of cells on the first PC during learning and attention. Values greater than the axis limits are pegged to the maximum displayed value.

(F) Average PSTHs of all recruited cells—in other words, cells that changed from non-selective to selective stimulus responses during learning; N = 332 and 263 cells recruited with preference for vertical stimulus or angled stimulus, respectively.

(G) Average PSTHs of all recruited cells during attention; N = 703 and 690 cells recruited with preference for vertical stimulus or angled stimulus, respectively. Shaded area represents SEM. Gray shading indicates 0–1 s window from stimulus onset used for analysis.

See also Figure S6.

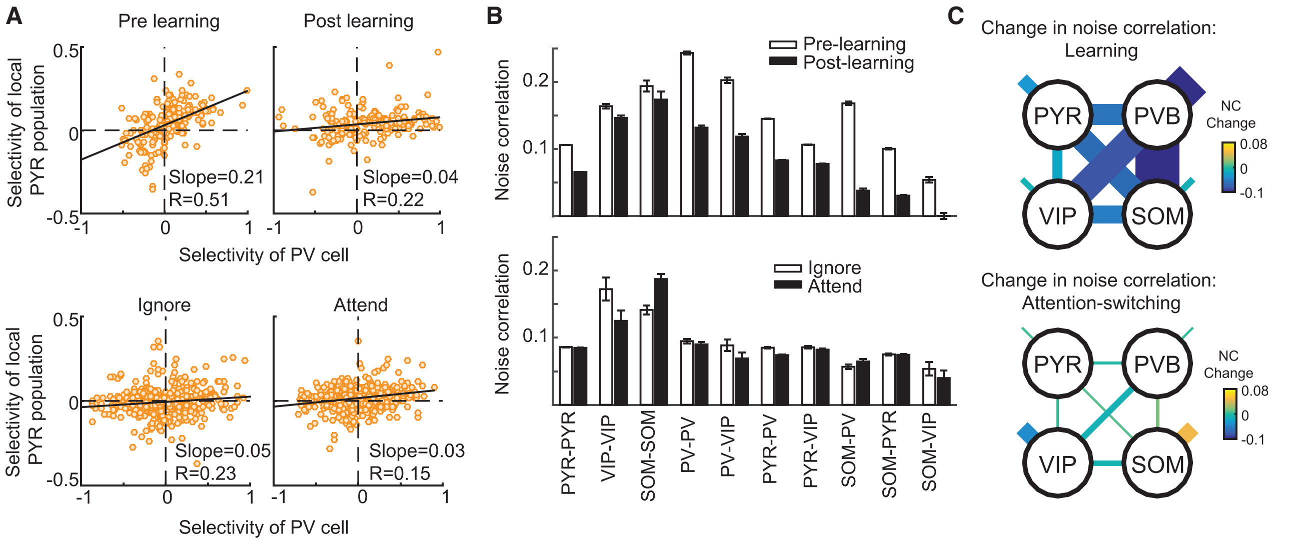

Figure 5. Distinct changes in interactions between excitatory and inhibitory cells during learning and attention

(A) Top, relationship between the selectivity of individual PV cells and the mean selectivity of the local PYR population within 100 μm of each PV cell, before (pre) and after (post) learning. N = 193 PV cells. Bottom, same comparison for the ignore and attend conditions of the attention-switching task. N = 427 PV cells.

(B) Average noise correlations between cell pairs belonging to the same or different cell classes, before and after learning (top) or in the ignore and attend conditions (bottom). Only cells with significant responses to the grating stimuli were included. The number of cell pairs in each cell class combination was as follows: pre-, post-learning, PYR-PYR 153,347, 84,119; VIP-VIP 1,519, 1,046; SOM-SOM 281, 128; PV-PV 2,935, 1,628; PV-VIP 1,390, 920; PV-PYR 36,652, 19,704; PYR-VIP 22,131, 4,368; SOM-PV 1,673, 798; SOM-PYR 11,374, 6,158; SOM-VIP 771, 519. Ignore/attend conditions, PYR-PYR 57,179; VIP-VIP 58; SOM-SOM 380; PV-PV 750; PV-VIP 126; PV-PYR 10,656; PYR-VIP 2,993; SOM-PV 792; SOM-PYR 6,354; SOM-VIP 134. Error bars represent SEMs. The full data distribution can be seen in Figure S5B.

(C) Changes in noise correlations (shown in B) due to learning (top) or attention (bottom) as indicated by line thickness and color code. Shorter line segments indicate change in noise correlations between cells of the same type.

See also Figure S5.

learning (Figure 5A, top, pre-learning, slope = 0.21, 95% confidence intervals [CIs] 0.16–0.26, R = 0.51, post-learning, slope = 0.04, CI 0.01–0.08, R = 0.22, bootstrap test for reduction in slope $p < 10^{-4}$), suggesting that during learning, PV cell activity became less dependent on the average stimulus preference of surrounding PYR cells. However, when we performed the same analysis comparing ignore and attend conditions, we found no difference in the correlation coefficient or slope of this relationship (Figure 5A, bottom, ignore, slope = 0.05, CI 0.03–0.07, R = 0.23, attend, slope = 0.03, CI 0.01–0.05, R = 0.15, bootstrap test for reduction in slope p = 0.06). The relationship appeared similar to that observed at the end of learning. This was despite the fact that PV cells displayed a comparable degree of selectivity increase with attention to that of learning.

To further explore the network signatures of changes during learning and attention, we computed noise correlations during the grating stimulus period between pairs of neurons within and across cell classes, before and after learning and during attend and ignore conditions. Since noise correlations are a measure of the stimulus-independent trial-to-trial co-variability of neural responses, they provide an estimate of mutual connectivity and shared inputs. As reported earlier, we found that during learning, SOM cells become de-correlated from pyramidal, PV, and VIP neurons, with the largest changes between cell classes (sign test, all reductions in noise correlation were significant at $p < 10^{-4}$ [Bonferroni corrected all $p < 10^{-3}$], with the exception of SOM-SOM cell pairs, p = 0.75, sign test [see also Khan et al., 2018]). Specifically, we observed a large reduction in noise correlation between SOM-PV, SOM-PYR, and SOM-VIP cell pairs during learning (Figures 5B and 5C, top, vertical grating stimulus; full distributions in Figure S5B).

In contrast, during attention switching, we found that the largest absolute changes in noise correlation were within cell classes, namely between SOM-SOM and VIP-VIP cell pairs (Figures 5B and 5C, bottom). SOM-SOM cell pairs displayed an increase in noise correlation (sign test, $p = 5 \times 10^{-10}$), whereas VIP-VIP pairs displayed decreased noise correlation (p = 0.02, Bonferroni corrected $p = 5 \times 10^{-9}$ and 0.2, respectively). In addition, PYR-PV and PV-PV cell pairs showed a significant reduction in noise correlation, although the absolute change was smaller ($p = 8 \times 10^{-19}$ and 0.03, Bonferroni corrected $p = 8 \times 10^{-18}$ and 0.3, respectively). Changes in running speed or licking could not account for the observed changes in noise correlations (Figures S4C and S4D).

Thus, learning and attention are associated with different patterns of changes in noise correlations between excitatory and multiple inhibitory cell classes, which is consistent with the idea that distinct mechanisms underlie these processes.

Modeling response changes during learning and attention

What changes in network properties underlie the observed changes during learning and attention? We recently developed a multivariate autoregressive (MVAR) linear dynamical system

CellPress
OPEN ACCESS

model to predict the activity of single cells based on interaction weights with their local neighbors. Analysis of the MVAR model fit to the neural responses during learning revealed that increased response selectivity after learning was associated with the reorganization of interaction weights between cells (Figures S7A–S7C; see also Khan et al., 2018). We tested whether similar changes in functional connectivity can account for the changes in stimulus responses observed with attention. We compared a model that allowed interaction weights to change across the attend and ignore conditions against a simpler model that used the same weights across both conditions. We found that the fit quality of the MVAR model, quantified by the cross-validated R^2, was actually lower for the model, allowing weights to change across the attend and ignore conditions, demonstrating that changing interaction weights during attention conferred no advantage to the model (Figure S7B). Even when weights were allowed to change in the MVAR model, we found stable PYR-PV interaction weights during attention, in contrast to the changes in weights observed during learning (Figure S7C). Together with the absence of reorganization of PYR-PV interactions during attention (Figure 5A, bottom), these results suggest that local functional connectivity is relatively stable during attention but changes during learning, possibly through long-term synaptic plasticity mechanisms.

Since the data-driven MVAR model analysis indicated that the selectivity changes were not predicted by changes in local functional interactions, we developed a detailed theoretical model of the local circuit enabling us to evaluate what type of external inputs could explain the attentional modulation of the local circuit. In this model, we represented each of the four cell types (PYR, PV, SOM, and VIP) by their population activity, corresponding to the average response across all cells with a given stimulus preference in the population. Population activity was determined by baseline activity, feedforward stimulus-related input, top-down attentional modulatory input, and connection weights with other cell populations (see Method details). The four neural populations were connected using experimentally derived connectivity values, similar to Kuchibhotla et al. (2017) (Figure 6A). The model's population responses resembled the average population stimulus responses of all four cell classes (Figure 6B, experimental responses shown in inset).

In the model, each population received fluctuations from cell-intrinsic sources (e.g., due to ion channel noise) and shared external sources (stimulus and top-down modulatory inputs; Figure 6A). The simulated noise correlations thus reflected both connectivity and fluctuations in the stimulus and modulatory inputs. Since functional connectivity weights between cell classes were stable across attend and ignore conditions, we modeled the changes in noise correlations during attention switching as arising from changes in the shared external fluctuations.

It is unclear whether attention has a multiplicative effect (Goris et al., 2014; Reynolds and Heeger, 2009) or an additive effect (Buracas and Boynton, 2007; Thiele et al., 2009). We therefore considered two different types of models with an additive or multiplicative effect of attentional modulation. We systematically simulated all of the conditions in which attentional modulation targeted different cell classes and combinations of cell classes. We then evaluated the stimulus selectivity changes and noise correlation changes induced by attentional modulation (Figure 6C). We looked for conditions that replicated our experimental findings, including that (1) attention increased only PYR and PV stimulus selectivity (Figure 2G) and (2) attention mainly increased SOM-SOM and decreased VIP-VIP noise correlations (Figure 5C, bottom). Of all of the conditions, only one matched both of these experimental findings, in which PYR and SOM cells received multiplicative attentional modulation (Figure 6C, arrows).

The model so far assumed equal influence of attentional modulation onto all cells. We next varied the relative strengths of modulation received by PYR and SOM cells to test whether the match to experimental findings could be improved. Specifically, the current model produced an increase in noise correlations between PYR-PYR, PYR-SOM, SOM-PV, and SOM-VIP cells, which was not observed experimentally. A model in which the attentional modulation of PYR was 0.7 times the modulation of SOM improved the match to the data (Figure S7D). This model replicated the increase in PYR and PV stimulus selectivity (Figure 6D) as well as the changes in SOM-SOM and VIP-VIP noise correlations, with only minor changes in noise correlations between other cell types (Figure 6E). Thus, a model in which PYR and SOM populations received different degrees of multiplicative attentional modulation best accounted for the changes in selectivity and noise correlations observed in the data (Figure S7E).

DISCUSSION

We show that improvements in sensory coding arising from learning or attention rely on distinct mechanisms, based on three lines of evidence. First, at the single-cell level, the effects of learning and attention are uncorrelated. Second, distinct patterns of firing rate changes underlie the increases in selectivity during learning and attention. Third, learning and attention are associated with different changes in functional interactions between cell classes. Our computational models suggest that learning relies on the reorganization of interactions in the local circuit, whereas attention relies on multiplicative top-down signals that target specific cell-classes.

Subpopulations of excitatory neurons modulated by learning and attention

Learning and attention are closely linked: attended objects are preferentially learned, and learning can bias the allocation of attention (Gilbert et al., 2000; Vartak et al., 2017). Although we show that learning and attention both lead to a similar increase in stimulus selectivity on average in PYR and PV cells, these increases are not driven by the same subset of neurons. Importantly, this does not mean that cells are either modulated by learning or attention. Instead, learning and attention each modulate the same neurons to varying degrees, and a neuron's degree of modulation during learning is uncorrelated with its degree of modulation by attention.

The basis of neural susceptibility to either learning- or attention-related modulations is poorly understood. For example, it may be related to intrinsic excitability (Brebner et al., 2020), expression of immediate-early genes (e.g., CREB [Han et al.,

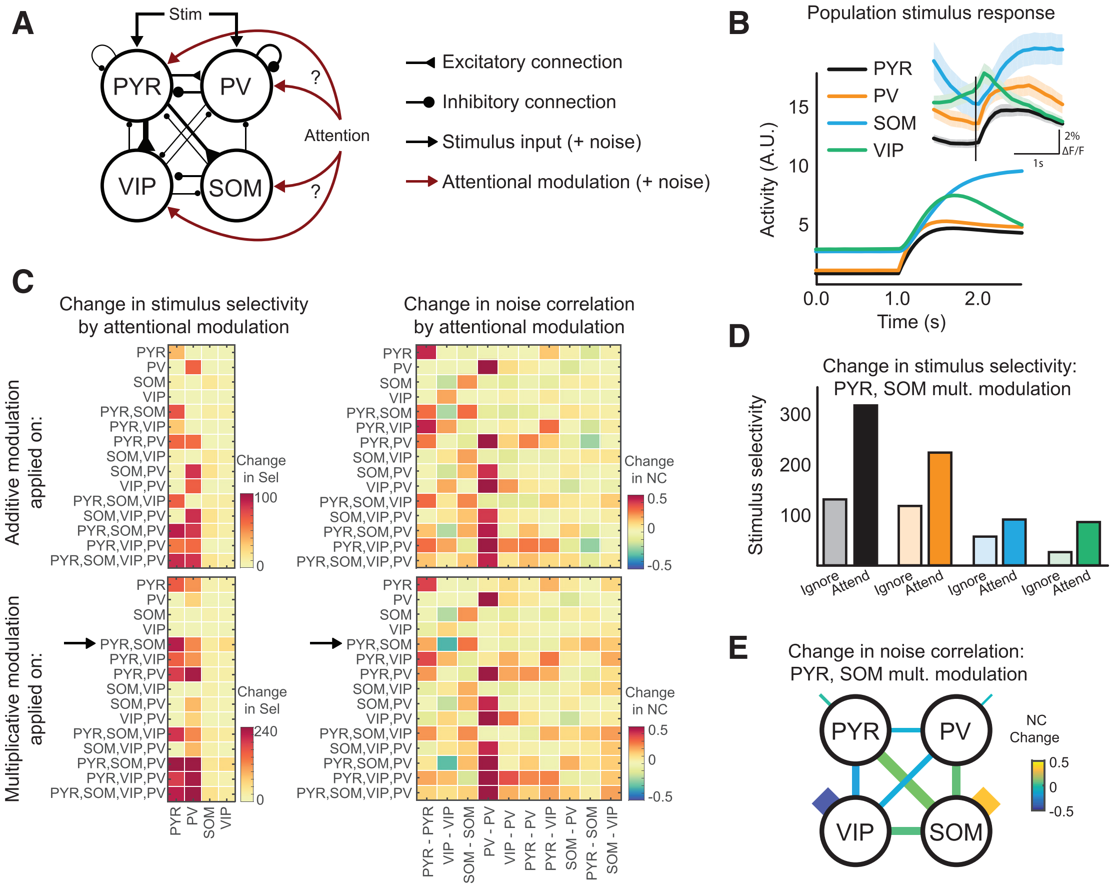

Figure 6. A circuit model can distinguish between different patterns of top-down attentional modulation

(A) The model architecture, indicating connectivity between different cell classes and possible sources of shared external fluctuations.

(B) Simulated responses of the 4 cell types to the preferred stimulus. Inset: experimentally obtained average responses of all of the cells in each cell class aligned to the vertical grating stimulus onset. Shading indicates SEM.

(C) Changes in stimulus selectivity and noise correlations (NCs) obtained from models with attentional modulation applied to different combinations of cell populations. Both additive and multiplicative modulations were tested. The arrow indicates the condition that best replicated the experimental changes in selectivity and noise correlation.

(D) Absolute selectivity of different cell classes without (ignore) and with (attend) attentional modulation provided to PYR and SOM populations, with PYR receiving 0.7 times the modulation of SOM (see Figures S7D and S7E).

(E) Changes in noise correlations (NC change) with attentional modulation as in (D) between and within the 4 cell classes, as indicated by line thickness and color code.

See also Figure S7.

2007] or Arc [Gouty-Colomer et al., 2016]; see also Holtmaat and Caroni, 2016), and pre- or post-synaptic expression of neuromodulator receptors (Disney et al., 2007; Herrero et al., 2008) or connectivity with distal and top-down inputs (Iacaruso et al., 2017; Marques et al., 2018). Our results impose an important restriction: these molecular or circuit mechanisms must be independent or exert a minimal influence on each other, since the effects of learning and attention on individual cells are uncorrelated.

While we have studied the three major classes of interneurons in the cortex (Xu et al., 2010), each of these classes contains further subdivisions of cell types (Tasic et al., 2016). Further studies may reveal functional differences between these subclasses describing their specific roles in learning and attention.

Suppression and enhancement of stimulus responses

We find that learning and attention lead to distinct patterns of suppression and enhancement of firing rates. Learning was dominated by selective suppression of responses to the non-preferred stimulus, perhaps because it is metabolically more efficient for implementing long-term selectivity changes (Howarth et al., 2012). Previous studies of associative conditioning have described both suppression and enhancement of responses in the sensory cortex (Gdalyahu et al., 2012; Goltstein et al., 2013; Makino and Komiyama, 2015). By longitudinally tracking the same neurons, we find that learning is largely accompanied by sparsification of cortical responses. Attention, in contrast, largely led to selectivity changes through selective enhancement of responses. This is consistent with a large body of work showing that the enhancement of attended responses is a

CellPress
OPEN ACCESS

common form of attentional modulation (McAdams and Maunsell, 1999; Speed et al., 2020; Spitzer et al., 1988; Wilson et al., 2019). Here, by studying the same neural population across both learning and attention, we demonstrate that V1 neurons are remarkably versatile, capable of displaying either selective enhancement or selective suppression of stimulus responses according to the current behavioral demand.

Changes in interactions

Imaging the activity of multiple cell classes simultaneously allowed us to investigate both interactions within and between excitatory and inhibitory cell classes. We found changes in interactions at two levels.

First, we observed a reorganization of interaction weights between PYR and PV cells during learning, possibly through long-term synaptic plasticity, which was captured quantitatively by a linear dynamical systems model. In contrast, attention did not lead to a similar change in interaction weights, suggesting that the short timescale of attention does not permit large-scale reorganization of connectivity patterns.

Second, we found changes in noise correlations between pairs of the same or different cell classes. Changes in noise correlations have been implicated in improved behavioral abilities during learning and attention (Jeanne et al., 2013; Ni et al., 2018). We found that noise correlation changes were dramatically different across learning and attention. Learning was marked by reductions in inter-cell class correlations. Specifically, SOM cells became decorrelated from the rest of the network. This transition potentially facilitates plasticity in the network by reducing the amount of dendritic inhibition from SOM cells that coincides with visual responses in excitatory cells (Khan et al., 2018). In contrast, attention changed the correlations of SOM-SOM and VIP-VIP cell pairs, leaving inter-cell class correlations relatively unchanged. Our model demonstrates that these changes can be explained by top-down input in the absence of local connectivity changes. Importantly, this relies on specific connectivity motifs across cell classes (Fino and Yuste, 2011; Hofer et al., 2011; Jiang et al., 2015; Pfeffer et al., 2013).

To account for the increased stimulus selectivity and noise correlation changes, we tested a variety of circuit architectures (Prinz et al., 2004). Top-down attentional modulation signals can be multiplicative (Goris et al., 2014; Reynolds and Heeger, 2009) or additive (Buracas and Boynton, 2007; Thiele et al., 2009), and they can target specific cell classes (Leinweber et al., 2017; Zhang et al., 2014, 2016). Here, the experimental results limited possible model architectures to a single one, with multiplicative top-down modulation targeting SOM and PYR cells. Top-down projections with specific targeting have been proposed to be central to the gating of plasticity, allowing attention to guide learning (Roelfsema and Holtmaat, 2018). Our predictions of targeted top-down projections provide a basis for future experimental work.

In summary, learning and attention lead to similar increases in neural response selectivity, but the effects are driven by different subsets of cells. Cells undergo distinct patterns of activity changes to achieve increased neural response selectivity during learning and attention. These results highlight the remarkable versatility by which a cortical circuit implements computations across short and long timescales.

STAR★METHODS

Detailed methods are provided in the online version of this paper and include the following:

- KEY RESOURCES TABLE
- RESOURCE AVAILABILITY
  - Lead contact
  - Materials availability
  - Data and code availability
- EXPERIMENTAL MODEL AND SUBJECT DETAILS
  - Animals and two-photon calcium imaging
- METHOD DETAILS
  - Behavioral training
  - Immunohistochemistry and image registration
  - Data analysis
  - Linear multivariate autoregressive system model
  - Circuit model
  - Additive model
  - Multiplicative model
  - Connectivity

SUPPLEMENTAL INFORMATION

Supplemental information can be found online at https://doi.org/10.1016/j.neuron.2021.11.016.

ACKNOWLEDGMENTS

We thank the GENIE Program and Janelia Research Campus of the Howard Hughes Medical Institute for making GCaMP6 material available. This work was supported by the European Research Council (S.B.H., HigherVision 337797; T.D.M.-F., NeuroV1sion 616509), the SNSF (S.B.H., 31003A_169525; A.G.K., PZ00P3_168046), EMBO (A.B., ALTF 74-2014), the Wellcome Trust (A.G.K., 206222/Z/17/Z; J.P., 211258/Z/18/Z; C.C., 200790/Z/16/Z; T.D.M.-F. and S.B.H., 090843/F/09/Z), the BBSRC (C.C., BB/N013956/1 and BB/N019008/1; A.G.K., BB/S015809/1), the EPSRC (C.C., EP/R035806/1), the Simons Foundation (C.C., 564408; M.S., SCGB 323228 and 543039), the Gatsby Charitable Foundation (M.S., T.D.M.-F., and S.B.H., GAT3361), the DFG (KAW 398005926), and Biozentrum core funds (University of Basel).

AUTHOR CONTRIBUTIONS

J.P., T.D.M.-F., S.B.H., and A.G.K. designed the experiments. J.P. and A.G.K. performed the experiments and analyzed the data. K.W. developed and analyzed the circuit model, with supervision from C.C. A.C. developed and analyzed the MVAR model, with supervision from M.S. A.B. performed the immunostaining and contributed to the post hoc cell-matching procedure. All of the authors discussed the data. J.P. and A.G.K. wrote the article, with input from all of the authors.

DECLARATION OF INTERESTS

The authors declare no competing interests

Received: January 31, 2021
Revised: July 31, 2021
Accepted: November 15, 2021
Published: December 13, 2021

REFERENCES

Brebner, L.S., Ziminski, J.J., Margetts-Smith, G., Sieburg, M.C., Reeve, H.M., Nowotny, T., Hirrlinger, J., Heintz, T.G., Lagnado, L., Kato, S., et al. (2020). The Emergence of a Stable Neuronal Ensemble from a Wider Pool of Activated Neurons in the Dorsal Medial Prefrontal Cortex during Appetitive Learning in Mice. J. Neurosci. *40*, 395–410.

Buracas, G.T., and Boynton, G.M. (2007). The effect of spatial attention on contrast response functions in human visual cortex. J. Neurosci. *27*, 93–97.

Chalupa, L.M., Coyle, R.S., and Lindsley, D.B. (1976). Effect of pulvinar lesions on visual pattern discrimination in monkeys. J. Neurophysiol. *39*, 354–369.

Chen, Y., Martinez-Conde, S., Macknik, S.L., Bereshpolova, Y., Swadlow, H.A., and Alonso, J.-M. (2008). Task difficulty modulates the activity of specific neuronal populations in primary visual cortex. Nat. Neurosci. *11*, 974–982.

Chen, T.-W., Wardill, T.J., Sun, Y., Pulver, S.R., Renninger, S.L., Baohan, A., Schreiter, E.R., Kerr, R.A., Orger, M.B., Jayaraman, V., et al. (2013). Ultrasensitive fluorescent proteins for imaging neuronal activity. Nature *499*, 295–300.

Chen, S.X., Kim, A.N., Peters, A.J., and Komiyama, T. (2015). Subtype-specific plasticity of inhibitory circuits in motor cortex during motor learning. Nat. Neurosci. *18*, 1109–1115.

Cohen, M.R., and Maunsell, J.H. (2009). Attention improves performance primarily by reducing interneuronal correlations. Nat. Neurosci. *12*, 1594–1600.

Desimone, R., and Duncan, J. (1995). Neural mechanisms of selective visual attention. Annu. Rev. Neurosci. *18*, 193–222.

Disney, A.A., Aoki, C., and Hawken, M.J. (2007). Gain modulation by nicotine in macaque v1. Neuron *56*, 701–713.

Fino, E., and Yuste, R. (2011). Dense inhibitory connectivity in neocortex. Neuron *69*, 1188–1203.

Froemke, R.C. (2015). Plasticity of cortical excitatory-inhibitory balance. Annu. Rev. Neurosci. *38*, 195–219.

Gdalyahu, A., Tring, E., Polack, P.-O., Gruver, R., Golshani, P., Fanselow, M.S., Silva, A.J., and Trachtenberg, J.T. (2012). Associative fear learning enhances sparse network coding in primary sensory cortex. Neuron *75*, 121–132.

Gilbert, C.D., and Li, W. (2012). Adult visual cortical plasticity. Neuron *75*, 250–264.

Gilbert, C.D., and Li, W. (2013). Top-down influences on visual processing. Nat. Rev. Neurosci. *14*, 350–363.

Gilbert, C., Ito, M., Kapadia, M., and Westheimer, G. (2000). Interactions between attention, context and learning in primary visual cortex. Vision Res. *40*, 1217–1226.

Goltstein, P.M., Coffey, E.B.J., Roelfsema, P.R., and Pennartz, C.M.A. (2013). In vivo two-photon Ca2+ imaging reveals selective reward effects on stimulus-specific assemblies in mouse visual cortex. J. Neurosci. *33*, 11540–11555.

Goris, R.L.T., Movshon, J.A., and Simoncelli, E.P. (2014). Partitioning neuronal variability. Nat. Neurosci. *17*, 858–865.

Gouty-Colomer, L.A., Hosseini, B., Marcelo, I.M., Schreiber, J., Slump, D.E., Yamaguchi, S., Houweling, A.R., Jaarsma, D., Elgersma, Y., and Kushner, S.A. (2016). Arc expression identifies the lateral amygdala fear memory trace. Mol. Psychiatry *21*, 364–375.

Gu, Y., Liu, S., Fetsch, C.R., Yang, Y., Fok, S., Sunkara, A., DeAngelis, G.C., and Angelaki, D.E. (2011). Perceptual learning reduces interneuronal correlations in macaque visual cortex. Neuron *71*, 750–761.

Han, J.-H., Kushner, S.A., Yiu, A.P., Cole, C.J., Matynia, A., Brown, R.A., Neve, R.L., Guzowski, J.F., Silva, A.J., and Josselyn, S.A. (2007). Neuronal competition and selection during memory formation. Science *316*, 457–460.

Herrero, J.L., Roberts, M.J., Delicato, L.S., Gieselmann, M.A., Dayan, P., and Thiele, A. (2008). Acetylcholine contributes through muscarinic receptors to attentional modulation in V1. Nature *454*, 1110–1114.

Hofer, S.B., Ko, H., Pichler, B., Vogelstein, J., Ros, H., Zeng, H., Lein, E., Lesica, N.A., and Mrsic-Flogel, T.D. (2011). Differential connectivity and response dynamics of excitatory and inhibitory neurons in visual cortex. Nat. Neurosci. *14*, 1045–1052.

Holtmaat, A., and Caroni, P. (2016). Functional and structural underpinnings of neuronal assembly formation in learning. Nat. Neurosci. *19*, 1553–1562.

Howarth, C., Gleeson, P., and Attwell, D. (2012). Updated energy budgets for neural computation in the neocortex and cerebellum. J. Cereb. Blood Flow Metab. *32*, 1222–1232.

Iacaruso, M.F., Gasler, I.T., and Hofer, S.B. (2017). Synaptic organization of visual space in primary visual cortex. Nature *547*, 449–452.

Jeanne, J.M., Sharpee, T.O., and Gentner, T.Q. (2013). Associative learning enhances population coding by inverting interneuronal correlation patterns. Neuron *78*, 352–363.

Ji, X.-Y., Zingg, B., Mesik, L., Xiao, Z., Zhang, L.I., and Tao, H.W. (2016). Thalamocortical Innervation Pattern in Mouse Auditory and Visual Cortex: Laminar and Cell-Type Specificity. Cereb. Cortex *26*, 2612–2625.

Jiang, X., Shen, S., Cadwell, C.R., Berens, P., Sinz, F., Ecker, A.S., Patel, S., and Tolias, A.S. (2015). Principles of connectivity among morphologically defined cell types in adult neocortex. Science *350*, aac9462.

Kanashiro, T., Ocker, G.K., Cohen, M.R., and Doiron, B. (2017). Attentional modulation of neuronal variability in circuit models of cortex. eLife *6*, e23978.

Karmarkar, U.R., and Dan, Y. (2006). Experience-dependent plasticity in adult visual cortex. Neuron *52*, 577–585.

Kato, H.K., Gillet, S.N., and Isaacson, J.S. (2015). Flexible Sensory Representations in Auditory Cortex Driven by Behavioral Relevance. Neuron *88*, 1027–1039.

Khan, A.G., Poort, J., Chadwick, A., Blot, A., Sahani, M., Mrsic-Flogel, T.D., and Hofer, S.B. (2018). Distinct learning-induced changes in stimulus selectivity and interactions of GABAergic interneuron classes in visual cortex. Nat. Neurosci. *21*, 851–859.

Kuchibhotla, K.V., Gill, J.V., Lindsay, G.W., Papadoyannis, E.S., Field, R.E., Sten, T.A.H., Miller, K.D., and Froemke, R.C. (2017). Parallel processing by cortical inhibition enables context-dependent behavior. Nat. Neurosci. *20*, 62–71.

Lee, S.-H., Kwan, A.C., Zhang, S., Phoumthipphavong, V., Flannery, J.G., Masmanidis, S.C., Taniguchi, H., Huang, Z.J., Zhang, F., Boyden, E.S., et al. (2012). Activation of specific interneurons improves V1 feature selectivity and visual perception. Nature *488*, 379–383.

Leinweber, M., Ward, D.R., Sobczak, J.M., Attinger, A., and Keller, G.B. (2017). A Sensorimotor Circuit in Mouse Cortex for Visual Flow Predictions. Neuron *95*, 1420–1432.e5.

Letzkus, J.J., Wolff, S.B.E., Meyer, E.M.M., Tovote, P., Courtin, J., Herry, C., and Lüthi, A. (2011). A disinhibitory microcircuit for associative fear learning in the auditory cortex. Nature *480*, 331–335.

Li, W., Piëch, V., and Gilbert, C.D. (2008). Learning to link visual contours. Neuron *57*, 442–451.

Makino, H., and Komiyama, T. (2015). Learning enhances the relative impact of top-down processing in the visual cortex. Nat. Neurosci. *18*, 1116–1122.

Marques, T., Nguyen, J., Fioreze, G., and Petreanu, L. (2018). The functional organization of cortical feedback inputs to primary visual cortex. Nat. Neurosci. *21*, 757–764.

McAdams, C.J., and Maunsell, J.H. (1999). Effects of attention on orientation-tuning functions of single neurons in macaque cortical area V4. J. Neurosci. *19*, 431–441.

Mitchell, J.F., Sundberg, K.A., and Reynolds, J.H. (2007). Differential attention-dependent response modulation across cell classes in macaque visual area V4. Neuron *55*, 131–141.

Mitchell, J.F., Sundberg, K.A., and Reynolds, J.H. (2009). Spatial attention decorrelates intrinsic activity fluctuations in macaque area V4. Neuron *63*, 879–888.

Ni, A.M., Ruff, D.A., Alberts, J.J., Symmonds, J., and Cohen, M.R. (2018). Learning and attention reveal a general relationship between population activity and behavior. Science *359*, 463–465.

CellPress
OPEN ACCESS

Pfeffer, C.K., Xue, M., He, M., Huang, Z.J., and Scanziani, M. (2013). Inhibition of inhibition in visual cortex: the logic of connections between molecularly distinct interneurons. Nat. Neurosci. *16*, 1068–1076.

Poort, J., Khan, A.G., Pachitariu, M., Nemri, A., Orsolic, I., Krupic, J., Bauza, M., Sahani, M., Keller, G.B., Mrsic-Flogel, T.D., and Hofer, S.B. (2015). Learning Enhances Sensory and Multiple Non-sensory Representations in Primary Visual Cortex. Neuron *86*, 1478–1490.

Prinz, A.A., Bucher, D., and Marder, E. (2004). Similar network activity from disparate circuit parameters. Nat. Neurosci. *7*, 1345–1352.

Reynolds, J.H., and Chelazzi, L. (2004). Attentional modulation of visual processing. Annu. Rev. Neurosci. *27*, 611–647.

Reynolds, J.H., and Heeger, D.J. (2009). The normalization model of attention. Neuron *61*, 168–185.

Roelfsema, P.R., and Holtmaat, A. (2018). Control of synaptic plasticity in deep cortical networks. Nat. Rev. Neurosci. *19*, 166–180.

Rutkowski, R.G., and Weinberger, N.M. (2005). Encoding of learned importance of sound by magnitude of representational area in primary auditory cortex. Proc. Natl. Acad. Sci. USA *102*, 13664–13669.

Sachidhanandam, S., Sermet, B.S., and Petersen, C.C.H. (2016). Parvalbumin-Expressing GABAergic Neurons in Mouse Barrel Cortex Contribute to Gating a Goal-Directed Sensorimotor Transformation. Cell Rep. *15*, 700–706.

Schoups, A., Vogels, R., Qian, N., and Orban, G. (2001). Practising orientation identification improves orientation coding in V1 neurons. Nature *412*, 549–553.

Snyder, A.C., Morais, M.J., and Smith, M.A. (2016). Dynamics of excitatory and inhibitory networks are differentially altered by selective attention. J. Neurophysiol. *116*, 1807–1820.

Speed, A., Del Rosario, J., Mikail, N., and Haider, B. (2020). Spatial attention enhances network, cellular and subthreshold responses in mouse visual cortex. Nat. Commun. *11*, 505.

Spitzer, H., Desimone, R., and Moran, J. (1988). Increased attention enhances both behavioral and neuronal performance. Science *240*, 338–340.

Tasic, B., Menon, V., Nguyen, T.N., Kim, T.K., Jarsky, T., Yao, Z., Levi, B., Gray, L.T., Sorensen, S.A., Dolbeare, T., et al. (2016). Adult mouse cortical cell taxonomy revealed by single cell transcriptomics. Nat. Neurosci. *19*, 335–346.

Thiele, A., Pooresmaeili, A., Delicato, L.S., Herrero, J.L., and Roelfsema, P.R. (2009). Additive effects of attention and stimulus contrast in primary visual cortex. Cereb. Cortex *19*, 2970–2981.

van Versendaal, D., and Levelt, C.N. (2016). Inhibitory interneurons in visual cortical plasticity. Cell. Mol. Life Sci. *73*, 3677–3691.

Vartak, D., Jeurissen, D., Self, M.W., and Roelfsema, P.R. (2017). The influence of attention and reward on the learning of stimulus-response associations. Sci. Rep. *7*, 9036.

Whitlock, J.R., Heynen, A.J., Shuler, M.G., and Bear, M.F. (2006). Learning induces long-term potentiation in the hippocampus. Science *313*, 1093–1097.

Wiest, M.C., Thomson, E., Pantoja, J., and Nicolelis, M.A.L. (2010). Changes in S1 neural responses during tactile discrimination learning. J. Neurophysiol. *104*, 300–312.

Williams, L.E., and Holtmaat, A. (2019). Higher-Order Thalamocortical Inputs Gate Synaptic Long-Term Potentiation via Disinhibition. Neuron *101*, 91–102.e4.

Wilson, A.M., Beck, J.M., and Glickfeld, L.L. (2019). Separable codes for read-out of mouse primary visual cortex across attentional states. bioRxiv. https://doi.org/10.1101/731398.

Wimmer, R.D., Schmitt, L.I., Davidson, T.J., Nakajima, M., Deisseroth, K., and Halassa, M.M. (2015). Thalamic control of sensory selection in divided attention. Nature *526*, 705–709.

Xiong, Q., Znamenskiy, P., and Zador, A.M. (2015). Selective corticostriatal plasticity during acquisition of an auditory discrimination task. Nature *521*, 348–351.

Xu, X., Roby, K.D., and Callaway, E.M. (2010). Immunochemical characterization of inhibitory mouse cortical neurons: three chemically distinct classes of inhibitory cells. J. Comp. Neurol. *518*, 389–404.

Yan, Y., Rasch, M.J., Chen, M., Xiang, X., Huang, M., Wu, S., and Li, W. (2014). Perceptual training continuously refines neuronal population codes in primary visual cortex. Nat. Neurosci. *17*, 1380–1387.

Yang, T., and Maunsell, J.H. (2004). The effect of perceptual learning on neuronal responses in monkey visual area V4. J. Neurosci. *24*, 1617–1626.

Yazaki-Sugiyama, Y., Kang, S., Câteau, H., Fukai, T., and Hensch, T.K. (2009). Bidirectional plasticity in fast-spiking GABA circuits by visual experience. Nature *462*, 218–221.

Zhang, S., Xu, M., Kamigaki, T., Hoang Do, J.P., Chang, W.-C., Jenvay, S., Miyamichi, K., Luo, L., and Dan, Y. (2014). Selective attention. Long-range and local circuits for top-down modulation of visual cortex processing. Science *345*, 660–665.

Zhang, S., Xu, M., Chang, W.-C., Ma, C., Hoang Do, J.P., Jeong, D., Lei, T., Fan, J.L., and Dan, Y. (2016). Organization of long-range inputs and outputs of frontal cortex for top-down control. Nat. Neurosci. *19*, 1733–1742.

STAR★METHODS

KEY RESOURCES TABLE

REAGENT or RESOURCE	SOURCE	IDENTIFIER
**Antibodies**		
Goat anti-parvalbumin	Swant	PVG-213; RRID AB_2650496
Mouse anti-parvalbumin	Swant	PV-235; RRID AB_10000343
Rabbit anti-Vasoactive intestinal peptide	ImmunoStar	Cat# 20077; RRID AB_572270
Rat anti-somatostatin	Millipore	MAB354; RRID AB_2255365
DyLight 405-AffiniPure Donkey Anti-Mouse	Jackson ImmunoResearch	Cat# 715-475-150; RRID AB_2340839
Rhodamine Red-X-AffiniPure Donkey Anti-Rabbit	Jackson ImmunoResearch	Cat# 711-295-152; RRID AB_2340613
Alexa Fluor 647-AffiniPure Donkey Anti-Rat	Jackson ImmunoResearch	Cat# 712-605-153; RRID AB_2340694
Alexa Fluor 594-AffiniPure Donkey Anti-Mouse	Jackson ImmunoResearch	Cat# 715-585-151; RRID AB_2340855
Alexa Fluor 647-AffiniPure Donkey Anti-Rabbit	Jackson ImmunoResearch	Cat# 711-605-152; RRID AB_2492288
DyLight 405-AffiniPure Donkey Anti-Rat	Jackson ImmunoResearch	Cat# 712-475-153; RRID AB_2340681
DyLight 405-AffiniPure Donkey Anti-Goat	Jackson ImmunoResearch	Cat# 705-475-147; RRID AB_2340427
**Bacterial and virus strains**		
AAV2.1-syn-GCaMP6f-WPRE	Addgene	Cat#100837
**Experimental models: organisms/strains**		
Mouse: C57BL/6	Biozentrum animal facility	N/A
Mouse: Rosa-CAG-LSL-tdTomato (JAX: 007914) crossed with PV-Cre (JAX: 008069)	Jackson Laboratory	JAX: 007914; RRID IMSR_JAX:007914<br>JAX: 008069; RRID IMSR_JAX:008069
Mouse: Rosa-CAG-LSL-tdTomato (JAX: 007914) crossed with VIP-Cre (JAX: 010908)	Jackson laboratory	JAX: 007914; RRID IMSR_JAX:007914<br>JAX: 010908; RRID IMSR_JAX:010908
**Software and algorithms**		
MATLAB	Mathworks	https://ww2.mathworks.cn/products/matlab.html; RRID: SCR_001622
Fiji (ImageJ)	NIH	https://imagej.net/software/fiji
Circuit model	Custom code	10.5281/zenodo.5674688

RESOURCE AVAILABILITY

Lead contact

Further information and requests for resources and reagents should be directed to and will be fulfilled by the lead contact Jasper Poort (jp816@cam.ac.uk).

Materials availability

This study did not generate new unique reagents.

Data and code availability

All data reported in this paper will be shared by the lead contact upon request. All original code has been deposited at https://zenodo.org/record/5674688 and is publicly available as of the date of publication. DOIs are listed in the Key resources table. Any additional information required to reanalyze the data reported in this paper is available from the lead contact upon request.

EXPERIMENTAL MODEL AND SUBJECT DETAILS

Experimental procedures for the behavioral task, surgery, two-photon calcium imaging, post hoc immunostaining and image registration have been described in detail in previous studies (Khan et al., 2018; Poort et al., 2015).

Animals and two-photon calcium imaging

All experimental procedures were carried out in accordance with institutional animal welfare guidelines and licensed by the UK Home Office and the Swiss cantonal veterinary office. Nine mice were used in this study, of which 7 were tracked across both learning and attention, one during learning alone and one during attention alone. Mice were C57BL/6 wild-type mice (3 males, 1 female, Janvier Labs), crosses between Rosa-CAG-LSL-tdTomato (JAX: 007914) and PV-Cre (JAX: 008069) (3 males), and crosses between Rosa-CAG-LSL-tdTomato and VIP-Cre (JAX: 010908) (1 male, 1 female) all obtained from Jackson Laboratory. Since we were able to retrieve cell class identity in all mice from the post hoc immunostaining (see below), the transgenically expressed tdTomato was rendered redundant. Data from these mice at pre and post learning data points were analyzed in a prior study (Khan et al., 2018). The data collected during the attention switching task has not been reported previously.

METHOD DETAILS

Mice aged P48-P58 were implanted with a chronic imaging window following viral injections of AAV2.1-syn-GCaMP6f-WPRE (Chen et al., 2013). Multi-plane two-photon imaging began approximately three weeks after surgery, during which 4 planes were imaged with 20 μm spacing at an imaging rate of 8 Hz for each imaging plane. Eight mice were imaged both pre-learning (either first or second day of training) and post-learning (either day 7, 8 or 9 of training), and during an attention switching task (1 session each, after 1 to 2 days of learning the attention switching task). Before each imaging session the same site was found by matching anatomical landmarks.

Behavioral training

Details of the behavioral task have been described in previous studies (Khan et al., 2018; Poort et al., 2015). Food restricted mice were trained in a virtual environment to perform a visual go-no go discrimination task. Trials were initiated by head-fixed mice running on a Styrofoam wheel for a randomly chosen distance in an approach corridor (black and white circle pattern unrelated to the task for 111cm followed by gray walls for 74-185 cm plus a random distance of gray walls chosen from an exponential distribution with mean 37 cm). Mice were then presented with either a vertical grating pattern (square wave gratings, 100% contrast) or an angled grating pattern (rotated 40° relative to vertical) on the walls of the virtual environment (grating corridor length 111 cm). In the vertical grating corridor, the mouse could trigger the delivery of a reward, a drop of soy milk, by licking the spout after it had entered a 'reward zone' a short distance (55.5 cm) into the grating corridor (mice often licked in anticipation of the reward zone). This was considered a 'hit' trial. If an animal did not lick by the end of the reward zone, this was considered a 'miss' trial. In the angled grating corridor, the mouse did not receive a reward, and a single lick or more in this corridor was considered a 'false alarm' trial. No punishment was given. Running through the angled corridor without licking was considered a 'correct rejection' trial. Mice typically stopped running when they licked the spout, visible as longer stays in in the grating corridor in the lick rasters (Figure S1). Mouse performance was quantified using a behavioral d-prime: $bd' = \Phi^{-1}(H) - \Phi^{-1}(F)$, where Φ^{-1} is the normal inverse cumulative distribution function, H is the rate of hit trials and F is the rate of false alarm trials.

After reaching high levels of discrimination performance, all mice were trained to switch between blocks of an olfactory and visual discrimination task (the attention switching task). This task is an attentional set-shifting task in which mice switch between two rules or attentional sets: either attending to and discriminating visual stimuli, or attending to and discriminating odor stimuli while ignoring the same visual stimuli. The visual blocks were the same as the visual discrimination task described above. In olfactory blocks, mice performed an olfactory go-no go discrimination task in which odor 1 (10% soya milk odor) was rewarded and odor 2 (10% soya milk with 0.1% limonene mixture) was not rewarded. Odors were delivered through a flow dilution olfactometer calibrated with a mini PID (Aurora) at 10%–20% saturated vapor concentration of the above solutions, and at 1 L/min flow rate. Before the presentation of odors, in 70% of randomly chosen trials mice were also presented with the same vertical or angled grating stimuli at different positions in the approach corridor, with the grating corridor ending before the onset of odors. Mice learnt to ignore these irrelevant grating stimuli while accurately discriminating the odors. On switching to the visual block, mice licked selectively to the rewarded grating as before. Block transitions were not explicitly cued and mice transitioned between the two rules by noticing changes in stimuli and reward contingencies. Mice typically performed two visual and two olfactory blocks in each session, data was pooled across blocks of the same type. After each block transition, we excluded trials in which the behavior of the mice was ambivalent (Poort et al., 2015). Each block typically contained 70-150 trials. Mice typically learnt to perform the attention switching task successfully within 1-2 days.

Immunohistochemistry and image registration

Brain fixation was performed by transcardial perfusion with 4% paraformaldehyde in phosphate buffer 0.1 M followed by 24 hours of post-fixation in the same solution at 4°C. The brains underwent two freeze-thaw cycles in liquid nitrogen, and were sliced tangentially to the surface of visual cortex. 80 μm slices were cut on a vibratome (Zeiss Hydrax V50) and were immunostained for PV, SOM and VIP (Khan et al., 2018). Primary and secondary antibodies are listed in the Key Resources Table. We imaged the slices with a confocal microscope (Zeiss LSM 700), and confocal z stacks were registered with the previously acquired *in vivo* imaging planes and z stacks of the recording sites. Cells were identified manually and assigned to cell classes based on immunostaining.

Data analysis

Regions of interest (ROIs) from motion-corrected image stacks were selected for each cell in each session. We adapted the method of Chen et al. (2013) to correct for neuropil contamination of calcium traces. Neuropil masks were created for each cell by extending the ROI by 25 μm and including all pixels that were more than 10 μm away from the cell boundary, excluding pixels assigned to other cells or segments of dendrites and axons (pixels that were more than 2 standard deviations brighter than the mean across all pixels in the neuropil mask). We performed a robust regression on the fluorescence values of the ROI and neuropil mask. We inspected the slope of this regression in a sample of our dataset and obtained a factor of 0.7 by which we multiplied the neuropil mask fluorescence (median subtracted) before subtracting it from the ROI fluorescence to obtain the neuropil-corrected raw fluorescence time series F(t). Baseline fluorescence $F_0(t)$ was computed by smoothing F(t) (causal moving average of 0.375 s) and determining for each time point the minimum value in the preceding 600 s time window. The change in fluorescence relative to baseline, ΔF/F, was computed by taking the difference between F and F_0, and dividing by F_0. The pre- and post-learning data was also used in Khan et al. (2018).

Responses were analyzed for the vertical and angled grating corridor by aligning neuronal activity to the onset of the stimuli. We used a Wilcoxon rank-sum test to determine if the response of a cell (average ΔF/F in a time window of 0-1 s after grating onset) was significantly different between vertical and angled gratings ($p < 0.05$). We used a Wilcoxon signed-rank test to determine if the response (ΔF/F 0-1 s) to the gratings significantly increased or decreased relative to baseline (−0.5 to 0 s). For visualizing stimulus-evoked responses and for computing the change in stimulus-evoked responses with learning and attention, we subtracted the pre-stimulus baseline (−0.5 to 0 s before stimulus onset) from the average response.

The selectivity of each cell was quantified as the selectivity index (SI), the difference between the mean response (0-1 s) to the vertical and angled grating divided by the pooled standard deviation, which was positive or negative for cells that preferred the vertical or angled grating respectively. We took the average of the absolute selectivity of all cells to obtain an average measure of the selectivity across a population of cells (including vertical and angled preferring cells). Cells were classified as significantly selective or non-selective based on whether their responses to the two grating stimuli in a time window of 1 s after grating onset were significantly different (Wilcoxon rank-sum test, $p < 0.05$). Recruited cells were all cells non-selective in the pre-learning/ignore condition and significantly selective in the post-learning/attend condition. PSTHs of recruited cells were averaged and the percentage change of responses was calculated in the 0-1 s window after stimulus onset, with negative values indicating reduced responses. In Figures 4F and 4G we selected cells on the basis of this selectivity change, which does not constrain the direction of the response change. We calculated the selectivity of the local PYR population around each PV cell by averaging the responses of all PYR cells, within 100 μm distance, to the two grating stimuli. Confidence intervals were calculated by a bootstrap procedure where we randomly selected cells with replacement 10,000 times to obtain the 2.5 and 97.5 percentiles. The P value was given by the percentage of bootstrapped pre-learning or ignore condition slope values that were lower than the post-learning or attend slope multiplied by two (two-sided test). To compute Δselectivity during learning and attention, we took the difference $SI^{post} - SI^{pre}$ or $SI^{attend} - SI^{ignore}$ for cells with positive selectivity post learning or in the attend condition. Similarly, we took the difference $-(SI^{post} - SI^{pre})$ or $-(SI^{attend} - SI^{ignore})$ for cells with negative selectivity post learning or in the attend condition.

To compute noise correlation, we first subtracted for each trial and each cell the average stimulus evoked responses across all trials. We then used the Pearson correlation coefficient to quantify the correlation between responses of pairs of cells. Changes in noise correlations with learning and attention between different cell types were tested using a sign test on all cells imaged pre- and post-learning or in the ignore and attend conditions.

In a previous study based on the learning dataset used here, we controlled for the effects of running and licking on neural responses (Khan et al., 2018). Here we performed similar analysis on the attention dataset. We controlled for the possible effect of variations in running speed across the ignore and attend conditions on stimulus selectivity and noise correlations using a stratification approach. We selected a subset of trials with similar distributions of running speed in the ignore and attend condition for each stimulus. We then recomputed the stimulus selectivity and noise correlations in the attend and ignore conditions and obtained similar results with and without stratification (Figures S4A and S4C). On excluding trials with licks in the analysis window (0-1 s after grating onset), we also obtained similar results for stimulus selectivity and noise correlations (Figures S4B and S4D).

Linear multivariate autoregressive system model

Details of the MVAR model are described in a previous study (Khan et al., 2018). We fit the activity of all simultaneously imaged neurons using a multivariate autoregressive (MVAR) linear dynamical system incorporating stimulus-related input, the simultaneously measured co-fluctuations from multiple cells of different cell types and the mouse running speed. We estimated the interaction weights between pairs of cells which describe the relationship between the activity of one cell and the activity of another cell at previous time points, conditioned over the activity of all other cells and over behavioral and sensory variability.

The learning-related data was previously studied in detail using this model (Khan et al., 2018). Here we fit the model separately to the learning and attention switching tasks, in each case fitting either separate interaction weights for the pre/post learning or ignore/attend conditions or a single set of weights to account for activity in both conditions. The different MVAR models were compared using leave-one-out cross validation (Figure S7B), measuring prediction quality on held-out data. We held out one vertical grating trial from the post learning or attend condition in the test set, using the remaining trials of all types for training. The MVAR model was fit to these training data, and the error in the model prediction was calculated for each time sample in the test trial. This procedure was repeated, leaving out each vertical grating trial in turn. We calculated an R^2 value for each cell combining errors across all of these trials. Specifically, the R^2 was defined relative to a baseline model which incorporated only the trial-averaged response profile of each cell, i.e., $R^2 = 1 -$ (sum of

squared errors in MVAR prediction)/(sum of squared errors in the trial-averaged response profile prediction). Running speed was not included in the model for the cross-validation analysis to facilitate comparison with alternative models. To determine whether the results from this analysis were influenced by differences in the goodness of fit, or degree of overfitting of the MVAR model to the learning and attention datasets, we estimated the degree of overfitting as the difference between the train and test R^2 values. We obtained similar distributions of overfitting in the learning and attention data by excluding sessions from the attention data with higher or lower overfitting estimates (14 of 29 sessions excluded from attention data, learning data left unchanged. After excluding these sessions, overfitting was not significantly different between learning and attention, p = 0.16, t test). The MVAR model fit to this subset of data produced the same results as Figure S7B, the attention data was better fit when the interaction weights were held fixed rather than free (Cross-validated $R^2 = 0.26 \pm 0.007$ weights free and 0.30 ± 0.007 weights fixed, $p = 3.34 \times 10^{-6}$).

Circuit model

We modeled a circuit consisting of an excitatory population PYR, and three inhibitory populations, corresponding to PV, SOM, and VIP interneurons. The activity of the population i is described by its calcium response r_i, which evolves over time according to one of the following equations:

Additive model

$$\tau_i \frac{dr_i}{dt} = -r_i + \phi\left(I_i^b + I_i^s + I_i^{TD} + \sum_j W_{ij} r_j + \sigma_i \cdot \left(\sqrt{\chi_i^{FF}}\,\xi_{FF}(t) + \sqrt{\chi_i^{TD}}\,\xi_{TD}(t) + \sqrt{1-\chi_i^{TD}-\chi_i^{FF}}\,\xi_i(t)\right)\right)$$

Multiplicative model

$$\tau_i \frac{dr_i}{dt} = -r_i + \phi\left(I_i^{TD}\left(I_i^b + I_i^s\right) + \sum_j W_{ij} r_j + \sigma_i \cdot \left(\sqrt{\chi_i^{FF}}\,\xi_{FF}(t) + \sqrt{\chi_i^{TD}}\,\xi_{TD}(t) + \sqrt{1-\chi_i^{TD}-\chi_i^{FF}}\,\xi_i(t)\right)\right),$$

where $i, j \in \{PYR, PV, SOM, VIP\}$ and
τ_i is the time constant of population i.
I_i^b is the baseline input to population i,
I_i^s is the stimulus-dependent feedforward input to population i,
I_i^{TD} is the modulatory top-down input - the attentional modulation of population i, and
$\sum_j W_{ij} r_j$ is the recurrent input from the local circuit and W_{ij} is the effective synaptic weight.

As in earlier models (Kanashiro et al., 2017), each population received private and shared noise. $\xi_i(t)$ is noise, private to each population, corresponding to noise arising from ion channels, or the activation function.

$\xi_{TD}(t)$ and $\xi_{FF}(t)$ are shared noise terms arising from shared modulatory top-down and/or feedforward inputs. ξ_i (t),$\xi_{TD}(t)$, and $\xi_{FF}(t)$ are drawn from a Gaussian distribution with zero mean and unit variance. We assume that external noise sources contribute equally.

$\phi(x)$ is the activation function:

$$\phi(x) = \begin{cases} 0 & \text{if } x<0 \\ (r_{max} - r_0)\tanh(x/(r_{max} - r_0)) & \text{if } x \geq 0 \end{cases}$$

PYR and PV populations receive an input current I_i^s upon presentation of their preferred stimulus (Ji et al., 2016) representing thalamic inputs. They receive a fraction of this input current ($0.2 \cdot I_s$) upon presentation of their non-preferred stimulus. Similar results were observed when SOM and VIP populations also received the same input current as PV cells. All populations received a constant baseline current input I_i^b. Each modulated population i received a top-down modulation I_i^{TD}, which took one of two values $\{x_{ignore}, x_{attend}\}$ depending on the absence or presence of attention (see Tables A and B). $r_0 = 1.0$ and $r_{max} = 20.0$ denote the minimum and maximum activity, respectively.

Table: Inputs to the multiplicative model. Shown are the values for the baseline, stimulus, and top-down inputs to the populations PYR, PV, SOM, and VIP. Top-down inputs depend on the condition, which is either ignore or attend: $\{x_{ignore}, x_{attend}\}$.

Population	baseline I_i^b	stimulus I_i^s	top-down I_i^{TD}
**PYR**	6.0	17.8	{1.0, 2.0}
**PV**	4.0	10.0	{1.0, 2.0}
**SOM**	1.2	0.0	{1.0, 2.0}
**VIP**	4.6	0.0	{1.0, 2.0}

Table: Inputs to the additive model. Shown are the values for the baseline, stimulus, and top-down inputs to the populations PYR, PV, SOM, and VIP. Top-down inputs depend on the condition, which is either ignore or attend: $\{x_{ignore}, x_{attend}\}$.

Population	baseline I_i^b	stimulus I_i^s	top-down I_i^{TD}
**PYR**	6.0	17.8	{0.0, 1.0}
**PV**	4.0	10.0	{0.0, 1.0}
**SOM**	1.2	0.0	{0.0, 1.0}
**VIP**	4.6	0.0	{0.0, 1.0}

We changed the contributions of noise sources to the overall noise in the populations, depending on the inputs population *i* received, according to Kanashiro et al. (2017). If population *i* received attentional modulation:

$$\chi_i^{TD} = \frac{1}{3}$$

otherwise:

$$\chi_i^{TD} = 0.$$

If population *i* received feedforward input:

$$\chi^{FF} = \frac{1}{3}$$

otherwise:

$$\chi^{FF} = 0.$$

The standard deviation of the total noise was given by:

$$\sigma_i = 0.5\sqrt{2}$$

Connectivity

We took the weight matrix *W* from Kuchibhotla et al. (2017), and adjusted only the baseline and stimulus inputs I_i^b and I_i^s such that the simulated neural responses matched the data.

$$W = \begin{pmatrix} W_{EE} & W_{EP} & W_{ES} & W_{EV} \\ W_{PE} & W_{PP} & W_{PS} & W_{PV} \\ W_{SE} & W_{SP} & W_{SS} & W_{SV} \\ W_{VE} & W_{VP} & W_{VS} & W_{VV} \end{pmatrix} = \begin{pmatrix} .017 & .956 & .512 & .045 \\ .8535 & .99 & .307 & .09 \\ 1.285 & 0 & 0 & .14 \\ 2.104 & .184 & .734 & 0 \end{pmatrix}$$

Each population was represented twice in the model, allowing us to measure noise correlations within cell classes.

We simulated the network without stimulus input for 5 s until the neural activity for each cell class reached steady state. Then we presented the non-preferred stimulus for 3 s, following which we waited another 4 s before we presented the preferred stimulus for 3 s. The simulation time step was 1 ms. We repeated this protocol for 100 trials. τ_{PYR} was 800 ms and τ_i with $i \in \{SOM, VIP, PV\}$ was 400 ms.

To calculate the selectivity of cell populations in the model, we subtracted the mean activity to the non-preferred stimulus $\overline{x}_N$ from the mean activity to the preferred stimulus $\overline{x}_P$ during 1 s after stimulus onset and normalized by their pooled standard deviation s_{pooled}:

$$SI = \frac{\overline{x}_P - \overline{x}_N}{s_{pooled}}$$

$$s_{pooled} = \sqrt{\frac{(n-1)s_P^2 + (n-1)s_N^2}{2n-2}}$$

where *n* is the number of trials, s_P is the standard deviation of the activity during the preferred stimulus, and s_N is the standard deviation of the activity during the non-preferred stimulus.

To determine the noise correlation between cell populations in the model, we calculated the average activity in populations *x* and *y* in each trial *i* in a 1 s time window after onset of the preferred stimulus: x_i and y_i. We calculated the means $\overline{x}$ and $\overline{y}$ and standard

CellPress
OPEN ACCESS

deviations σ_x and σ_y of the activity over trials for each population. We then calculated noise correlations between populations x and y over $n = 100$ trials according to the following equation:

$$NC_{xy} = \frac{1}{n-1}\sum_{i=1}^{n}\left(\frac{x_i - \overline{x}}{\sigma_x}\frac{y_i - \overline{y}}{\sigma_y}\right).$$

For Figure S7D, I_{PV}^{TD} and I_{VIP}^{TD} were 0.0, and we varied I_{SOM}^{TD} continuously between 1 and 2.2 and I_{PYR}^{TD} proportionally to I_{SOM}^{TD} as indicated in the figure.

Neuron, Volume *110*

Supplemental information

Learning and attention increase visual response selectivity through distinct mechanisms

Jasper Poort, Katharina A. Wilmes, Antonin Blot, Angus Chadwick, Maneesh Sahani, Claudia Clopath, Thomas D. Mrsic-Flogel, Sonja B. Hofer, and Adil G. Khan

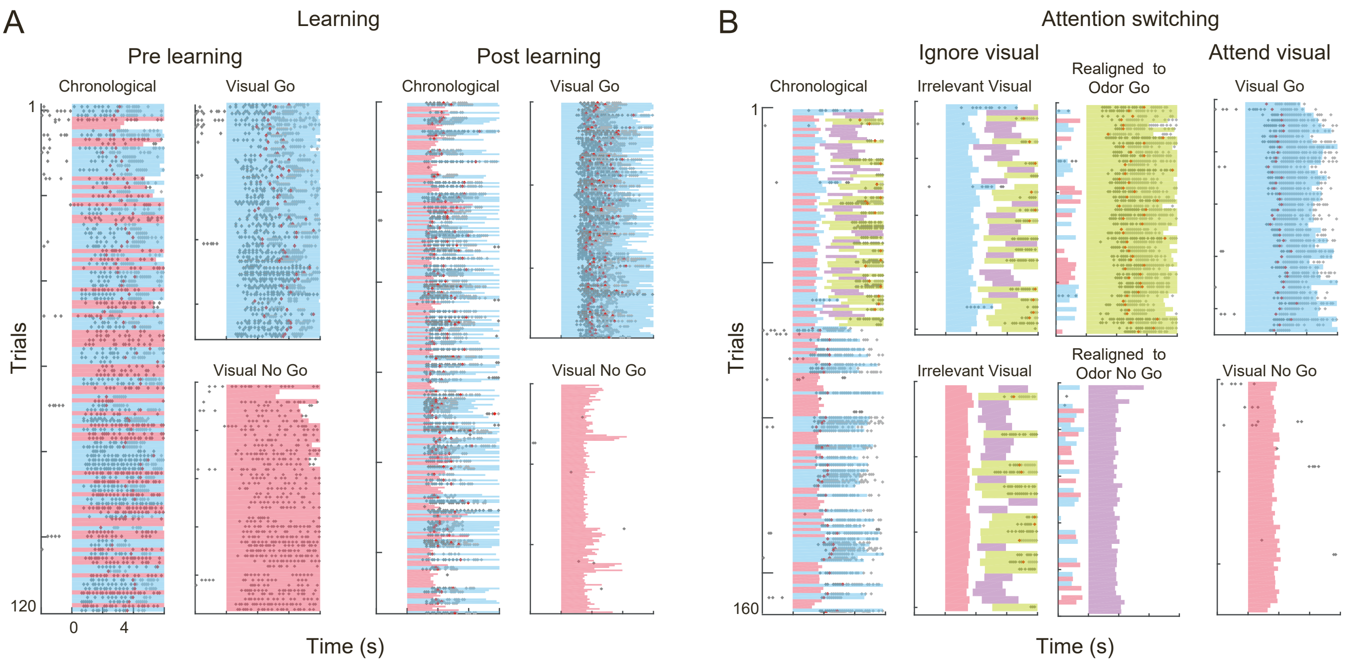

Figure S1. Example behavior sessions (Related to Figure 1). A) Lick rasters from example sessions pre- and post-learning. B) Example session of attention switching task, one block each of ignore and attend visual stimuli. Each row is a trial aligned to stimulus onset, black dots indicate licks, red dots indicate reward delivery, red and blue shading indicates presence of vertical and angled visual grating stimuli respectively, green and purple indicates odor1 and odor2 delivery respectively.

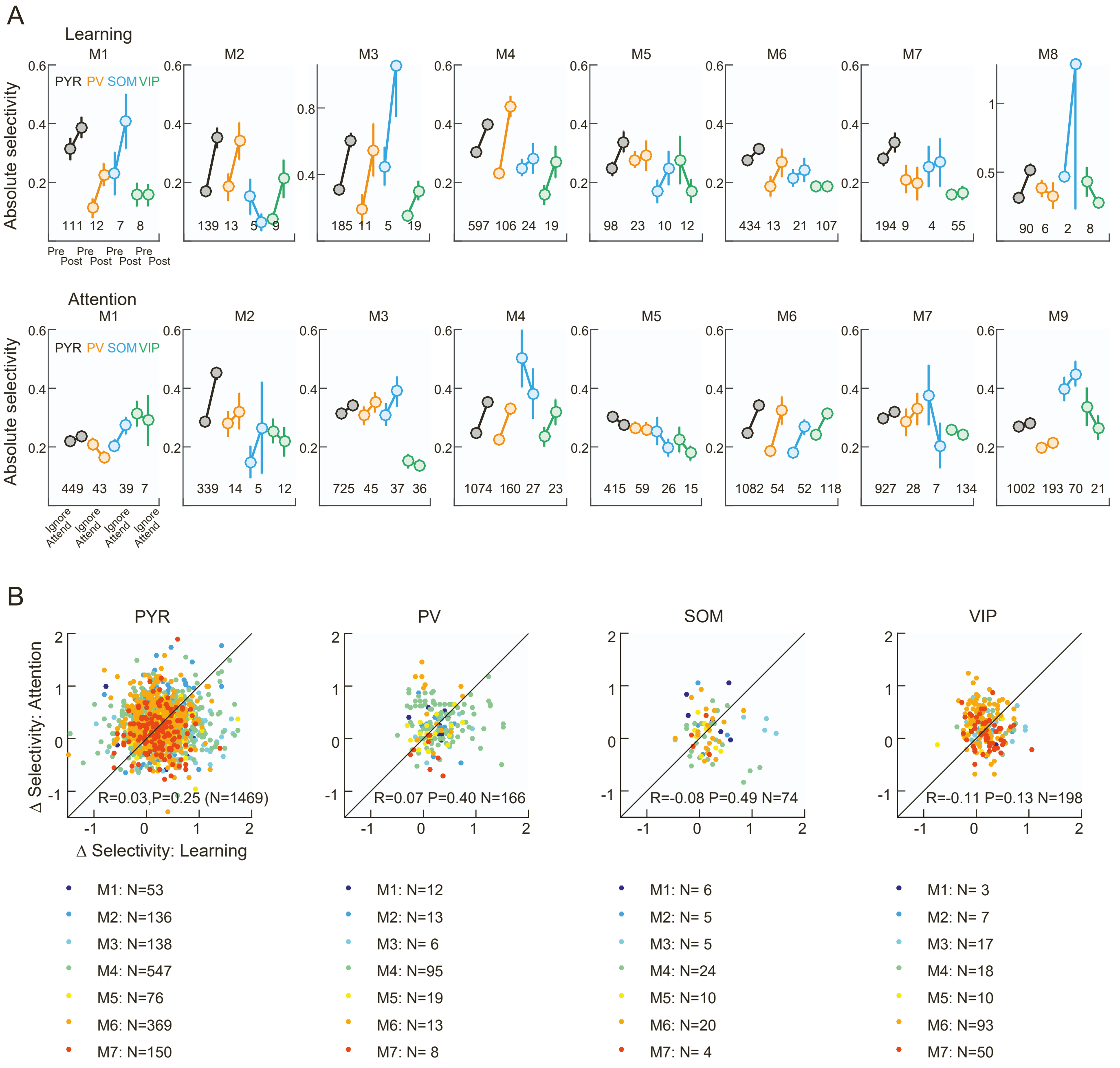

Figure S2. Data distributed across individual mice and cell classes (Related to Figure 2). A) Average absolute selectivity of the 4 cell classes before and after learning (top) and in the ignore and attend conditions (bottom) from all cells recorded from each mouse. Error bars represent SEM. Numbers indicate Ns for each cell class in each mouse. 7 mice were tracked across both learning and attention, one during learning alone and one during attention alone. B) Relationship between Δ Selectivity with learning and ΔSelectivity with attention for all cells tracked across both learning and attention from each mouse and each cell class. Colors indicate different mice, Ns indicate number of matched cells belonging to each cell class in each mouse.

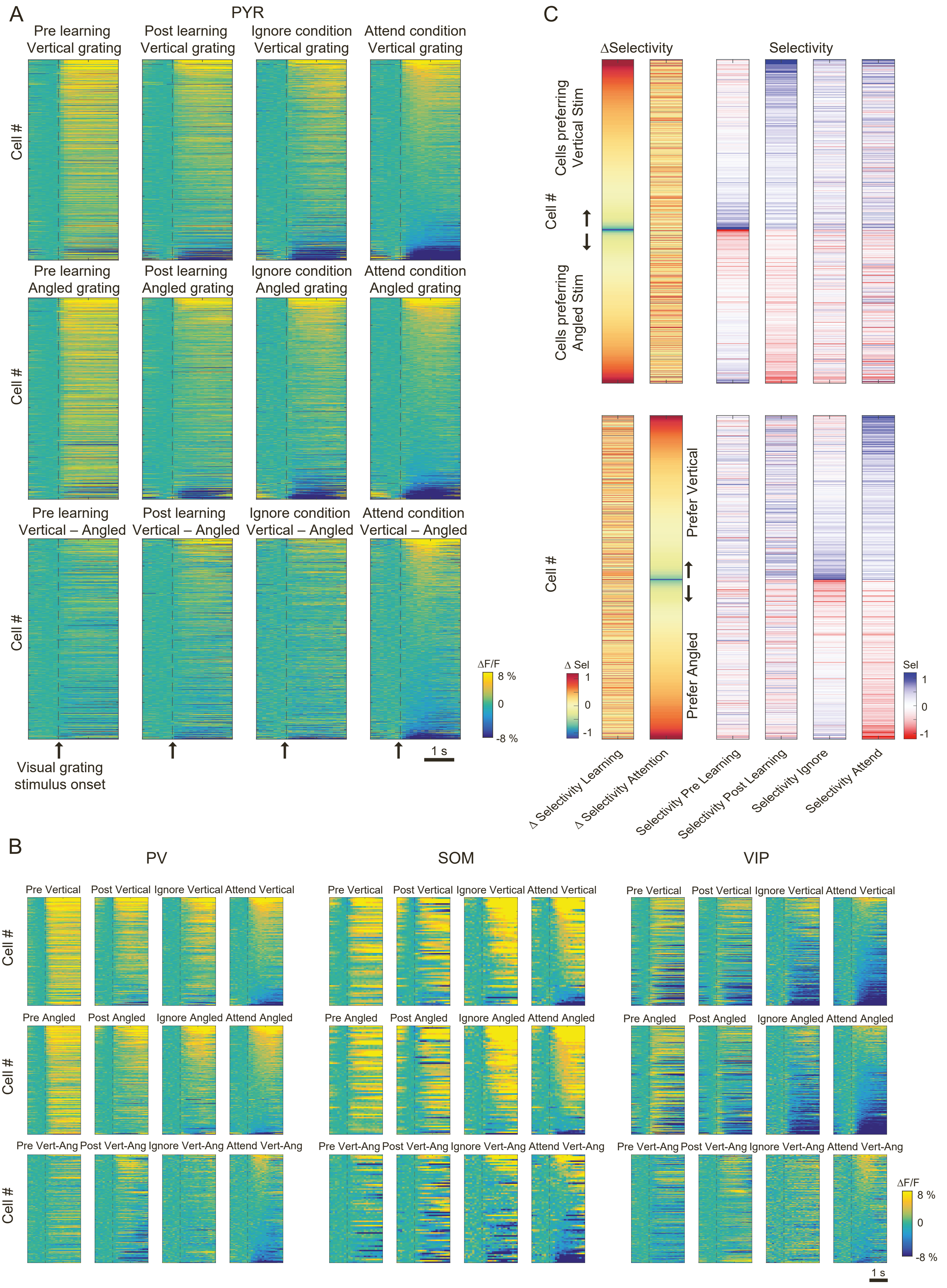

Figure S3. Responses and selectivity of cells across learning and attention switching (Related to Figure 3). A) Average responses of all PYR cells that were matched across the learning and attention tasks (N = 1469 cells). Responses are shown pre and post learning and in the ignore and attend conditions (columns). Responses are aligned to the vertical grating, angled grating and

the difference between the two (rows). Cells are sorted in the final column (attend condition) by their average response amplitude 0–1 s from stimulus onset, and the remaining three panels in the same row are shown with the same cell sorting, to aid comparing the same cells' responses in different conditions. All responses are baseline corrected (subtraction of baseline Δ F/F –0.5 to 0 s before stimulus onset) and aligned to grating onset (dashed line). B) Same as A) for the three interneuron classes, N = 166 PV cells, 74 SOM cells and 198 VIP cells. C) ΔSelectivity for the same cells during learning and attention displayed in color code (left, similar to Figure 3A). The same cell sorting is maintained throughout to show the selectivity of the same cells in the different conditions (right). Top and bottom are the same data sorted differently; cells are sorted by Δselectivity during learing (top) or attention (bottom), and by splitting the data into those cells which prefer vertical or angled stimuli in the post learning or attend condition respectively (indicated by arrows).

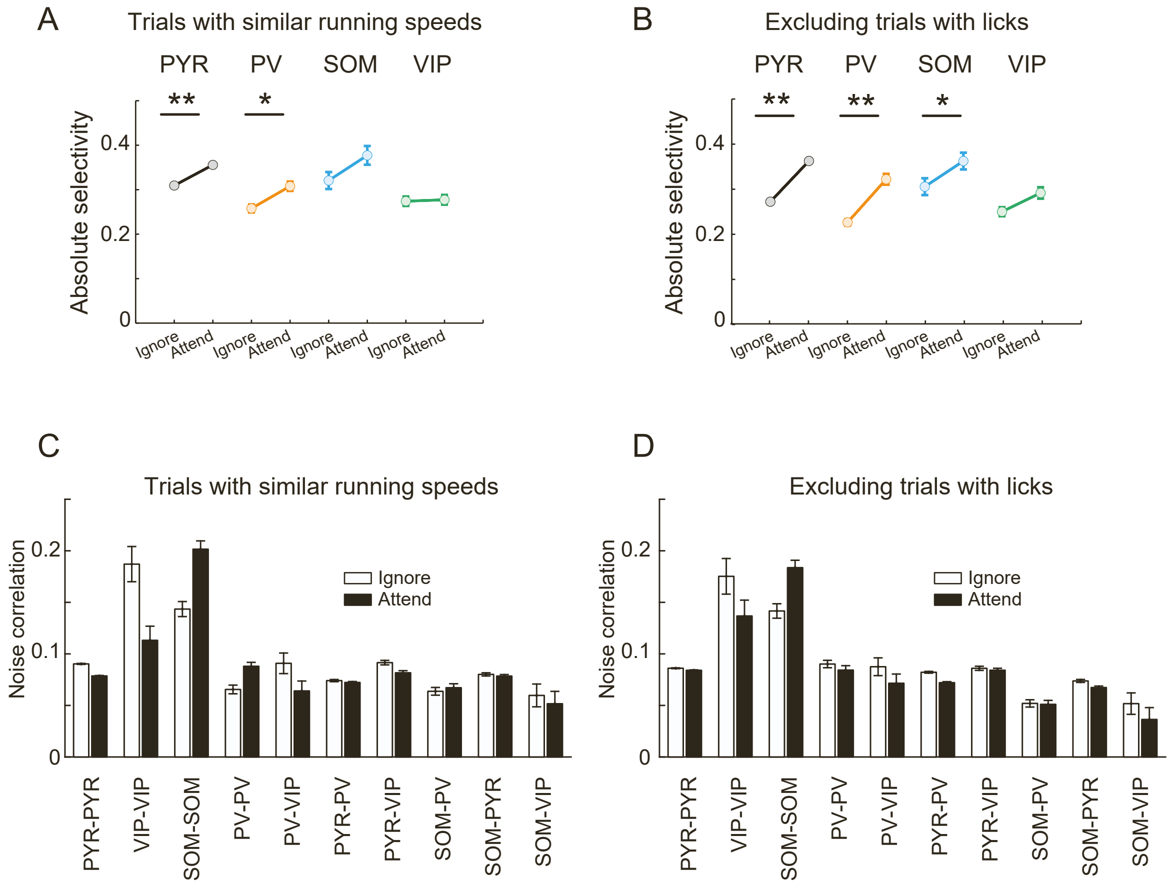

Figure S4. Differences in running speed and licking cannot account for the pattern of changes in stimulus selectivity and noise correlations (Related to Figures 2 and 5). A) Mean absolute selectivity of each cell class in the ignore and attend conditions (computed in the period of 0-1s after grating onset) after equalizing the distributions of running speed in the two conditions for each stimulus presentation. B) Mean absolute selectivity of each cell class when excluding all trials with licks. Sign test, **, $P < 0.001$; *, $P < 0.05$. C-D) Similar analysis for noise correlations measured during the vertical grating response (0-1 s from stimulus onset). Error bars represent SEM. Similar analysis was done on the learning dataset in Khan et al 2018 showing that changes in running speed and licking could not account for the pattern of changes in stimulus selectivity and noise correlations during learning.

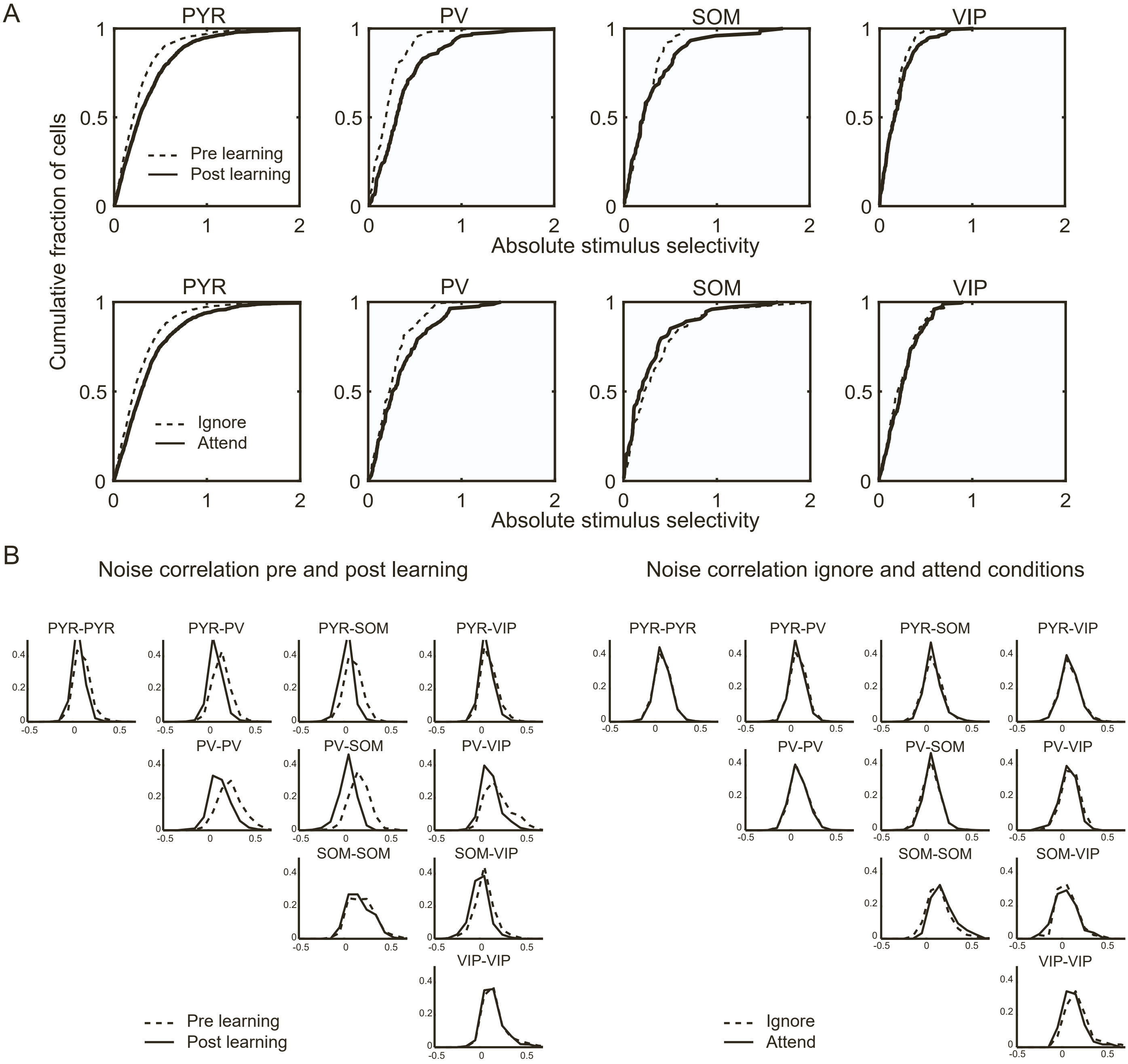

Figure S5. Distributions of selectivity and noise correlation (Related to Figures 2 and 5). A) Cumulative histograms of stimulus selectivity of each cell class. Selectivity was measured during the grating response (0-1 s from stimulus onset) before and after learning, and in the ignore and attend condition of the attention switching task. Cells were tracked both pre- and post-learning and during the attention task, N = 1469 PYR, 166 PV, 74 SOM and 198 VIP cells B) Distributions of noise correlation between cell pairs of each combination of cell classes during the vertical grating stimulus presentation. Noise correlation was measured during the grating response (0-1 s from stimulus onset) between cell pairs of each combination of cell classes, before and after learning (left), and in the ignore and attend condition of the attention switching task (right). The number of cell pairs in each cell class combination was as follows: pre-, post-learning, PYR–PYR 153347, 84119; VIP–VIP 1519, 1046; SOM–SOM 281, 128; PV–PV 2935, 1628; PV–VIP 1390, 920; PV–PYR 36652, 19704; PYR-VIP 22131, 4368; SOM–PV 1673, 798; SOM–PYR 11374, 6158; SOM–VIP 771, 519. Ignore/attend conditions, PYR–PYR 57179; VIP–VIP 58; SOM–SOM 380; PV–PV 750; PV–VIP 126; PV–PYR 10656; PYR–VIP 2993; SOM–PV 792; SOM–PYR 6354; SOM–VIP 134.

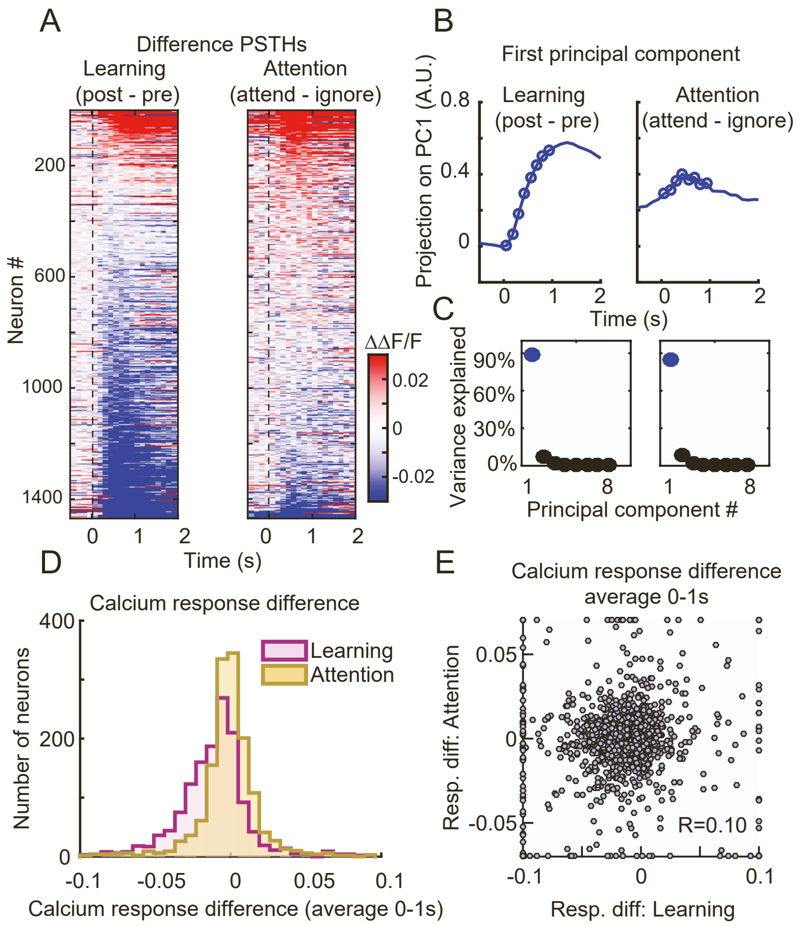

Figure S6. Response changes during learning and attention (Related to Figure 4). A-C) Similar to Figure 4A-C for non-rewarded angled stimulus. A) Difference in calcium responses to the non-rewarded angled stimulus, post minus pre learning (left) or attend minus ignore conditions (right) for all matched PYR cells (Difference-PSTHs). Responses are baseline corrected (subtraction of baseline ΔF/F –0.5 to 0 s before stimulus onset) and aligned to grating onset (dashed line). Cells are sorted by their average amplitude 0–1 s from stimulus onset. N = 1469 matched cells here and below. B) First principal component (PC) of the difference-PSTHs from the learning (left) and attention data (right). Circles indicate the time points (0-1s) used to determine the PCs. C) Percentage of variance explained by each PC during learning (left) and attention (right). D) Distribution of average calcium response difference (difference-PSTHs averaged 0-1s) in response to rewarded vertical grating stimulus, during learning and attention (P = 0, sign test). E) Relationship between the calcium response difference during learning and attention ($R = 0.10$, $P = 2.13 \times 10^{-4}$). Values greater than the axis limits are pegged to the maximum displayed value. Similar results as D and E were obtained with non-rewarded angled gratings, data not shown.

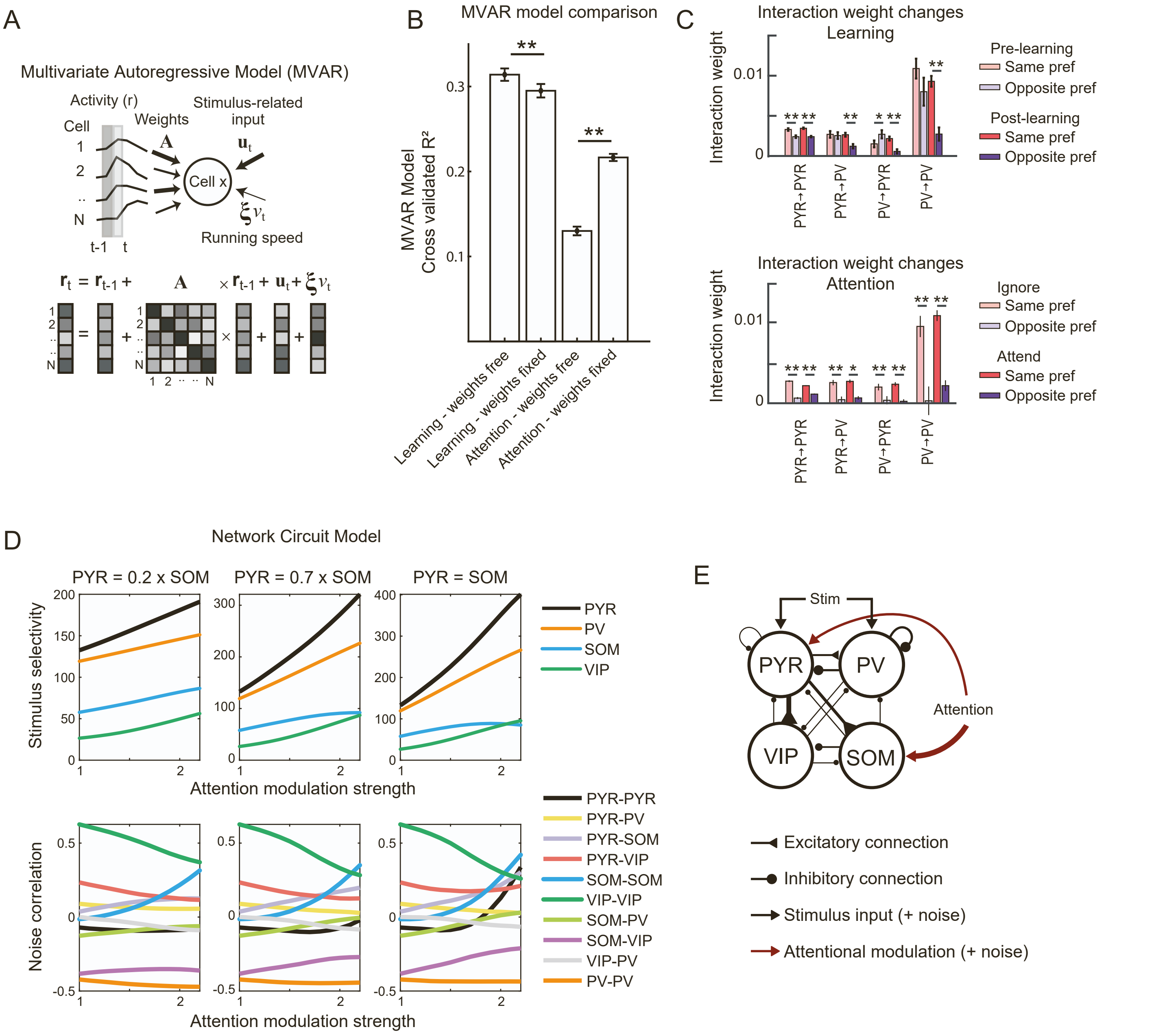

Figure S7. Computational modelling of learning and attention-related activity changes (Related to Figure 6). A) Schematic depicting the MVAR model which fits single-trial responses by estimating the contribution of stimulus-locked input, recurrent inputs from the local cell population and running speed. B) Comparison of different MVAR models. Cross-validated R^2 of different versions of the MVAR model fit to data with different constraints. When fitting pre- and post-learning data, cross-validated R^2 is higher when interaction weights are allowed to change from pre to post learning (learning: weights free, learning: weights fixed). When fitting attention data, cross-validated R^2 is lower when interaction weights are allowed to change between ignore and attend conditions (attention: weights free, attention: weights fixed). These results were unchanged when we matched the degree of overfitting in the learning and attention datasets, see Star Methods. $^*P < 0.05$ and $^{**}P < 10^{-3}$, here and below. C) In an MVAR model where weights were allowed to change, average interaction weights are shown for cell pairs of specific cell classes, and with the same or opposite stimulus-input preference before and after learning (top) or during ignore and attend conditions (bottom). Error bars indicate SEM. Stronger weights between same orientation preference pairs emerged during learning, and this pattern did not change with attention. D) Changes in stimulus selectivity (top) and noise correlation between cells (bottom) for varying degrees of attention modulation applied to SOM and PYR cells. Three combinations are shown with varying degrees of modulation applied to PYR relative to SOM populations. Left: model with PYR = 0.2×SOM modulation. Middle: model with PYR = 0.7×SOM modulation. Right: model with PYR = SOM modulation. Modulation of PYR and SOM populations with PYR = 0.7×SOM modulation best fits the data. E) Schematic showing the final circuit model which best accounts for the data.
